# Assessing Geographic Inequalities in Childhood Immunisation Coverage: A Critical Scoping Review of Spatial Analysis Methods

**DOI:** 10.3390/vaccines14070572

**Published:** 2026-06-29

**Authors:** Adrien Allorant, Nicole Bergen, M. Carolina Danovaro-Holliday, Joshua Lorin, Gustavo Caetano Corrêa, Danielle Boyda, Johanna Lee Belanger, Ravi Shankar Santhana Gopala Krishnan, Rocco Panciera, Ahmad Reza Hosseinpoor

**Affiliations:** 1 Department of Data, Digital Health, Analytics and AI, World Health Organization, CH-1211 Geneva, Switzerland; adrien.allorant@soton.ac.uk (A.A.); bergenn@who.int (N.B.); belangerj@who.int (J.L.B.); santhanagopalakr@who.int (R.S.S.G.K.); 2 Department of Social Statistics and Demography, Statistical Sciences Research Institute (S3RI), University of Southampton, Southampton SO17 1BJ, UK; 3 Department of Immunization, Vaccines and Biologicals, World Health Organization, CH-1211 Geneva, Switzerland; 4 Gavi, the Vaccine Alliance, CH-1211 Geneva, Switzerland; jlorin@gavi.org (J.L.); gus.correa@proton.me (G.C.C.); dboyda@gavi.org (D.B.); 5 Digital Health Transformation, Access and Equity Unit, UNICEF—Nairobi Centre of Excellence, Nairobi 00100, Kenya; rpanciera@unicef.org

**Keywords:** immunisation coverage, spatial analysis, small-area estimation, health inequality, scoping review

## Abstract

**Background:** Spatial analysis methods, including model-based geostatistics (MBG), small-area estimation (SAE), and cluster detection, are increasingly used to map subnational immunisation coverage and identify geographic inequalities in low- and middle-income countries. However, the extent to which these methods capture the multidimensional determinants of immunisation uptake, and whether their outputs inform programme decisions in practice, remains unclear. **Methods:** We conducted a critical scoping review following the Preferred Reporting Items for Systematic reviews and Meta-Analyses extension for Scoping Reviews guidelines, systematically searching PubMed and Google Scholar for studies applying spatial statistical methods to childhood immunisation coverage or equity. Findings were synthesised using a combination of descriptive summary and thematic and interpretive synthesis. **Results:** We included 50 studies from the 421 papers identified. Spatial methods have successfully revealed subnational coverage inequalities that national averages obscure, and studies developed in collaboration with national programme teams, integrating routine health system data alongside household surveys, produced the most operationally relevant outputs. However, most studies relied exclusively on survey data with a limited incorporation of supply-side determinants, and few discussed how uncertainty in estimates should constrain downstream use. Although a growing number of studies articulated clear implementation pathways, confirmed programmatic uptake of spatial outputs remained largely undocumented. The emergence of machine learning approaches (8 of 50 studies) offers predictive gains but introduces additional challenges around transparency and quality assurance for governance use. **Conclusions:** Spatial methods are becoming more frequently used for immunisation but are more likely to contribute to immunisation equity goals when co-produced with programme teams, matched to decision-relevant geographies, and accompanied by transparent documentation of model assumptions and limitations. Future research should prioritise quality frameworks for algorithm-assisted health estimates and systematic evaluation of whether spatial outputs improve decision-making relative to existing data sources.

## 1. Introduction

Childhood immunisation is one of the most effective public health interventions, averting an estimated 154 million deaths since the Expanded Programme on Immunisation was launched by the World Health Organisation (WHO) in 1974, the vast majority among children younger than five years old [1]. Yet, despite these achievements, vaccine-preventable diseases remain a significant cause of morbidity and mortality in many low- and middle-income countries (LMICs). This gap is often explained by persistent inequalities in coverage: in 2024, an estimated 14.3 million children globally received no routine vaccines (“zero-dose” children), with more than half concentrated in just nine countries. National averages can mask considerable subnational variation, and within-country inequalities frequently disadvantage populations in rural, conflict-affected, or socioeconomically poor areas [2]. These disparities in coverage reinforce the need for equity-focused approaches. Throughout this review, we distinguish between immunisation inequalities (observable differences in coverage across geographic areas or population groups) and inequities, which refer to differences that are unfair, avoidable, or remediable [3]. All inequities are inequalities, but not all inequalities are necessarily inequities: identifying a geographic pattern of low coverage is a measurement task, whereas judging that pattern to be unjust requires evidence about its social determinants and modifiability [4]. A central question of this review is: under what data and decision conditions can spatial methods produce coverage estimates that are good enough to inform real immunisation programme choices, rather than remaining analytically interesting but operationally unused?

Extensive research has shown that children who are unvaccinated or under-vaccinated tend to come from households marked by poverty, poor health-service access, and lower caregiver education [5,6,7,8]. Multiple conceptual frameworks have been proposed to explain the determinants of immunisation uptake. For example, Phillips et al. identified three principal domains influencing vaccine coverage: (i) caregivers’ intent to vaccinate, (ii) community access to vaccination (e.g., physical proximity, transportation, social support), and (iii) health facility readiness (e.g., the availability and quality of vaccination services) [9]. Similarly, the WHO’s Behavioural and Social Drivers of Vaccination framework outlines four broad domains that shape vaccination behaviours: what people think and how they feel about vaccines; social processes (including community norms and peer influence); motivation to vaccinate; and practical issues such as service availability and ease of access [10]. Other frameworks likewise emphasise factors related to caregivers and the community, the supply and quality of health services, and the broader systems or policy context [6,7,11]. Despite differences in terminology, these frameworks share common elements: they all highlight an interplay between demand-side factors (e.g., beliefs and attitudes), supply-side barriers (e.g., stockouts, facility-level constraints), and broader environmental determinants (e.g., geographic remoteness, local conflict). These frameworks illustrate that a child’s likelihood of receiving a vaccine depends on multiple intersecting domains: individual intent and confidence, social support and cultural norms, and the readiness of local health services to deliver vaccines.

A crucial dimension of these inequalities is geography. The communities most disadvantaged in immunisation coverage are often those that are remote, distant from functioning health services, or affected by conflict and poverty, and are concentrated particularly in sub-Saharan Africa, South Asia, and parts of Latin America. For these populations, where children live is closely linked to whether they are reached by vaccines [12]. Recognising this, global immunisation initiatives have made a clear push to “reach every child” through a geographic lens. WHO’s Immunisation Agenda 2030 (IA2030) and Gavi’s strategies, from Gavi 5.0 (2021–2025) to the current Gavi 6.0 (2026–2030), prioritise reaching zero-dose and under-immunised children, with geospatial approaches featuring prominently in both frameworks [13,14]. Specific tools envisaged include digital maps and micro-plans, satellite identification of settlements, and community e-registries [15]. While geography is not the sole determinant of under-immunisation, it provides an actionable framework for identifying and reaching populations affected by multiple intersecting vulnerabilities (including limited access to antenatal care, lower maternal education, and socioeconomic marginalisation) that tend to cluster in underserved areas [16]. IA2030’s and Gavi’s shared focus on subnational data use and geospatial tools implies not only better triangulation and visualisation of routine information for geographic targeting and decision-making but also a systematic use of spatial statistical methods to combine diverse data sources and generate immunisation coverage estimates at a much finer spatial resolution than traditional surveys or administrative reports allow. Advanced spatial analysis techniques can help map immunisation coverage and pinpoint pockets of under-vaccination that might otherwise remain hidden within national or regional statistics [5].

### 1.1. Spatial Analysis in Immunisation

When spatial analysis methods are applied to immunisation outcomes (e.g., zero-dose, single vaccine coverage, fully vaccinated), it cannot be automatically assumed that these models will capture the key dimensions of inequality underlying coverage gaps [17]. Many of the fundamental drivers of immunisation status, such as the provision and quality of local vaccination services, supply-chain reliability, caregivers’ risk perceptions, or cultural norms, are not easily represented [8,18] in the kinds of geospatial covariates typically used in these models (e.g., satellite-derived environmental features). Moreover, even when such factors are measurable, including them as covariates may be inappropriate if the resulting estimates are intended for downstream analyses, where these human factors would themselves be exposures of interest rather than predictors to adjust for. Recent research mapping other health outcomes (such as HIV prevalence and child mortality rates) using model-based geostatistics (MBG) has shown that for outcomes largely driven by human factors (behavioural, social, or health system factors), commonly available spatial covariates often have limited predictive power [19,20]. This raises questions about the adequacy of these models for immunisation, where human-mediated determinants dominate. At the same time, an emerging strand of geospatial work has begun to characterise how external shocks (including climate-related events, conflict displacement, and pandemic-era service interruptions) reshape the geographic distribution of under-vaccination over time [21], expanding the analytical agenda for spatial immunisation research beyond static cross-sectional mapping.

Beyond the question of statistical adequacy, there is also the challenge of how to translate granular coverage maps into immunisation policy or planning. High-resolution maps can provide a snapshot view of subnational inequalities where substantial spatial variation exists, but they typically come with wide uncertainty intervals, especially in data-sparse areas. This challenge may be particularly acute for immunisation, where coverage is typically measured among a subset of surveyed households with children aged 12–23 months, a sample that can be very small at the cluster level, amplifying uncertainty in local estimates. Furthermore, emerging evidence suggests that districts with higher prevalence of under-vaccination may also exhibit greater within-district heterogeneity [22], raising questions about whether district-level estimates, however precise, can reliably guide sub-district targeting where impact may be greatest. This uncertainty may limit their usefulness for finely tuning programmatic decisions or for tracking year-to-year changes in immunisation coverage at the local level. Thus, advanced spatial analyses can yield insights into where immunisation gaps might be located, but the apparent geographic precision of the maps can mask substantial statistical uncertainty, potentially limiting their reliability for policy or planning decisions relative to the data sources (surveys, administrative data) that countries currently use.

Consequently, the use of advanced spatial analysis methods for immunisation coverage raises two closely related challenges. First, to what extent do cluster detection (which identify statistically unusual concentrations of low coverage in observed survey data), SAE (which produces coverage estimates for administrative units such as districts by borrowing statistical strength across areas) and MBG (which produces continuous, high-resolution coverage surfaces by combining geo-referenced survey data with spatial covariates within a Bayesian framework) adequately capture the underlying social, behavioural, and health system factors that shape immunisation uptake? Second, how can national immunisation programmes, global health agencies, and local decision makers make evidence-informed use of these subnational coverage estimates for policy and planning? In particular, we must consider how these model-derived maps can inform both routine immunisation strengthening and supplementary immunisation activities, including their potential to identify susceptible populations and enable more precisely targeted campaigns and the pursuit of more equitable service delivery. Addressing these questions is critical to ensure that research efforts and innovative methodologies ultimately translate into equity-oriented policies and tangible health gains.

A wide range of spatial approaches has been developed to address these challenges, from simple cluster detection to hierarchical small-area models and fine-scale geostatistical mapping, often in combination with other statistical or machine learning techniques. Before turning to the evidence from our review, we briefly outline these main families of spatial methods as they appear in the current immunisation literature.

### 1.2. Overview of Spatial Analysis Approaches for Immunisation

A growing body of literature has focused on spatially characterising where under-immunisation occurs using household surveys and/or routinely collected programmatic data. Across the studies included in our review, the primary spatial approaches fell into a small number of recurring families rather than a long tail of distinct methods. For descriptive purposes, we group them into: (i) spatial cluster detection and autocorrelation analyses, which identify localised clusters or “cold spots” of low coverage; (ii) SAE and related hierarchical models, which generate estimates for administrative units by borrowing strength across geographic areas; (iii) MBG, which combines geo-referenced data and covariates to produce continuous, high-resolution surfaces of immunisation coverage; and (iv) a smaller set of combined or alternative approaches (e.g., machine learning models with spatial components). These groupings simply reflect the main families of methods we found in the immunisation literature, rather than an exhaustive taxonomy of spatial statistics.

Cluster detection methods (such as Moran’s I for global spatial autocorrelation, Local Indicators of Spatial Association (LISA) for local hot/cold spots, or Kulldorff’s spatial scan statistic) flag statistically significant clusters of under-vaccination [23]. In this context, “statistically significant” means that immunisation coverage values are more spatially clustered or dispersed than would be expected under spatial randomness, without saying anything about why those clusters exist or how they relate to other variables. The outputs of such analyses are typically maps highlighting specific survey clusters or local areas where coverage was significantly lower (or higher) than expected. Unlike MBG, cluster detection approaches do not produce estimates for unsampled locations and are purely descriptive of the observed data.

SAE techniques often employ hierarchical or multilevel models to improve the precision of subnational estimates by pooling information across domains (geographic areas, age-groups, etc.) [24]. These methods usually produce discrete coverage estimates for administrative units (e.g., districts or counties) rather than continuous surfaces often associated with MBG. Here, “continuous surface” refers to a fine regular grid (for example, 1 × 1 km pixels). In an SAE model, individual-level survey data are combined with area-level covariates and random effects (which may include spatially structured effects) to stabilise estimates for areas with small sample sizes.

The MBG framework leverages Bayesian spatial models to interpolate household survey data and produce fine-grained, continuous surfaces of immunisation coverage estimates, that is, discrete coverage values defined on a dense regular grid (e.g., 1 × 1 km pixels) across the study area. MBG extends classical geostatistical interpolation methods such as Kriging, which uses spatial autocorrelation among observed values to predict coverage at unsampled locations, by embedding them in a Bayesian framework that integrates covariates and propagates uncertainty through the full posterior distribution. Typical MBG workflows include: (i) assembling relevant environmental, demographic, or accessibility raster covariates; (ii) fitting a spatially structured Gaussian process to account for residual spatial autocorrelation; and (iii) generating posterior predictive surfaces at resolutions like 1 × 1 km or 5 × 5 km. The motivation for using MBG is generally to identify “micro-zones” of low coverage and geographic inequality with a high level of spatial detail [25].

Both SAE and MBG build on longstanding traditions in medical geography and disease mapping, leveraging survey data and covariates to interpolate or smooth estimates for unsampled locations, whereas cluster detection methods focus on revealing patterns present in the survey data itself [26]. A further advantage of SAE and MBG is that they provide explicit measures of statistical uncertainty (e.g., posterior intervals) for each estimate, which can, in principle, be carried forward and used in downstream decision-making [27].

From a programmatic perspective, the three approaches differ in important ways. SAE and cluster detection are, by design, constrained to the areal units used as input (for example, districts or provinces), whereas MBG produces gridded estimates that can be flexibly aggregated to any geographic unit and overlaid with other spatial data (such as other health programmes or environmental layers). Cluster detection methods do not model relationships with covariates and are therefore best suited to exploratory analyses that simply flag unusual spatial patterns. By contrast, SAE and MBG explicitly relate coverage to covariates, providing richer information on how immunisation coverage correlates with social, economic, or health system factors and thereby support more detailed modelling and policy analysis.

Cluster detection models and SAE and MBG spatial analysis techniques have become powerful tools for highlighting inequalities in immunisation coverage. High-resolution modelled maps from SAE or MBG are particularly useful under the current equity agenda: they can reveal localised areas of low coverage (potential “cold spots” of under-immunisation) that would be obscured by coarse aggregates, thereby highlighting communities most in need of intervention. Some of the first large-scale applications of SAE and MBG in public health occurred as part of global projects such as the Malaria Atlas Project, the Local Burden of Disease, and WorldPop [20,28,29,30,31,32], after which many researchers sought to replicate and refine these approaches for other diseases and settings. Efforts have also been dedicated to evaluating their predictive performances using statistical criteria. To date, the two systematic reviews published on the topic of spatial applications to health outcomes and coverage mapping were focused on technical aspects of these applications: specifically, the covariates used, and the type of validation techniques (e.g., in-sample vs. out-of-sample cross-validation) [33,34]. However, the literature to date has not meaningfully addressed the practical question of under what conditions these estimates provide substantially improved accuracy (i.e., closeness of estimates to true coverage values) and precision (i.e., width of uncertainty intervals) over direct survey estimates, nor has it examined the relative costs of generating geospatial estimates versus expanding survey sampling to achieve comparable subnational precision.

This critical scoping review, therefore, assesses how cluster detection, SAE and MBG, and a set of hybrid or alternative spatial approaches (e.g., GIS-based accessibility measures and machine learning models with spatial components) have been used to measure inequalities in immunisation coverage and to inform efforts to address resulting inequities. Drawing on existing frameworks for immunisation uptake and equity (such as Phillips et al.’s domains of intent, access and readiness, IA2030’s focus on zero-dose and missed communities, and Tanahashi’s effective coverage logic), we ask two main questions: (i) to what extent do different spatial methods capture key demand-side, supply/system and contextual determinants of vaccination, and (ii) how, if at all, have their outputs been used by policymakers and programme managers in practice? By integrating evidence from the methodological literature with insights on implementation and use, this review aims to inform the development of more effective, context-specific interventions to reduce gaps in vaccination coverage.

## 2. Methods

### 2.1. Study Design and Rationale for a Critical Scoping Review

Our aim in conducting a critical scoping review was to integrate a diverse body of literature on cluster detection, SAE and MBG methods, focusing on their relevance for measuring inequalities in childhood immunisation coverage, and to consider the practical applications of these measurements, adopting an explicitly interpretive lens. A standard systematic review would not have suited the conceptual diversity of this literature, which spans spatial statistics, epidemiology, and health systems [35]. Critical scoping reviews combine broad coverage with structured appraisal, building on the foundational scoping review framework of Arksey and O’Malley [36] and the methodological refinements proposed by Tricco and colleagues [37], incorporating critical interpretive synthesis-inspired features (iterative refinement of questions, constant comparison across diverse sources, and attention to theory use) to support a critical reading of methods and claims. In line with critical reviews, we sought to catalogue spatial methods applied to inequalities in childhood immunisation, and also to identify and interrogate the assumptions underlying various modelling approaches. Because key constructs related to spatial correlation and predictive covariates draw upon multiple fields, including economics, demography, epidemiology, and global health, this interpretive lens accommodated a broad range of studies that do not fit neatly within a more traditional systematic review. To strengthen the transparency and quality of our synthesis, we followed PRISMA-ScR (Preferred Reporting Items for Systematic reviews and Meta-Analyses extension for Scoping Reviews) guidance for scoping reviews in framing questions, eligibility, and reporting. The review question was scoped following the Population–Concept–Context (PCC) framework recommended for scoping reviews [37]: the population comprised infants and children eligible for routine childhood immunisation; the concept comprised spatial and geospatial statistical methods (cluster detection, SAE, MBG, and related approaches) applied to immunisation coverage or equity; and the context was any geographic setting, with no a priori restriction to low- and middle-income countries. Selection was purposive and iterative, typical of scoping reviews, with inclusion decisions based on conceptual relevance to our questions rather than exhaustiveness. The search strategy, inclusion criteria, and data-charting structure were documented before screening, and PRISMA-ScR conformance was maintained throughout (Table S1). We did not undertake a formal risk-of-bias appraisal or meta-analysis, and all counts are descriptive of our sample rather than the universe of studies.

### 2.2. Search Strategy and Selection of Evidence

The questions guiding this work, refined iteratively during the scoping process as is typical of scoping reviews, were: (i) to what extent do different spatial methods capture key demand-side, supply-system and contextual determinants of vaccination, and (ii) how have their outputs been used by policy makers and programme managers in practice?

In July 2024, we conducted comprehensive searches of PubMed and Google Scholar using a comprehensive set of predefined keywords covering spatial methods, immunisation, and inequities (full search strings for each database are provided in Section S1) restricted to publications from 2000 onward to reflect the widespread adoption of computer-based GIS systems, geocoded household surveys, and model-based spatial methods relying on digital covariates (e.g., satellite-derived data), rather than the historical origins of spatial analysis in public health. In total, this initial database search yielded 421 records. We then applied the inclusion criteria to focus the selection. Studies were included if they (i) reported a quantitative measure of childhood immunisation coverage (e.g., coverage percentage, coverage gaps) as an outcome, or (ii) employed a spatial analysis method as the primary analytical approach. We initially cast a broad net using generic spatial keywords, and during full-text review classified studies into three analytically dominant method families (cluster detection, small area estimation, and model-based geostatistics) based on the primary analytical strategy used. We excluded studies that (a) did not address childhood immunisation outcomes (e.g., studies focusing on other health outcomes using spatial methods); (b) lacked a spatial analysis method (e.g., studies using choropleth maps for visualisation only, or spatial autocorrelation tests solely as regression diagnostics); or (c) did not present primary data or analysis (e.g., systematic reviews, commentaries, meeting reports, or framework papers).

Applying these criteria, we identified 56 articles for full-text review. All records were imported into Covidence (Covidence systematic review software, Veritas Health Innovation, Melbourne, Australia) for deduplication and screening management. At the screening stage, one reviewer examined titles and abstracts for obvious relevance (objective screening), after which full texts were reviewed in depth. In the full-text review, we took a more interpretive approach to relevance: beyond the strict inclusion criteria, we considered each study’s conceptual contribution to our research questions. This meant that if a study only marginally fit the criteria but offered valuable insight into spatial inequalities or immunisation systems, we retained it. Conversely, studies that technically met inclusion criteria but provided minimal information relevant to our synthesis (for instance, a purely methodological paper with no discussion of immunisation equity implications) were excluded.

This stage was followed by a series of conversations with experts at WHO, UNICEF, and Gavi, the Vaccine Alliance, from September to December 2024, who provided grey literature and internal documents for review. These materials included unpublished technical reports and operational assessments that offered insight into the practical uptake (or non-uptake) of geospatial outputs, information largely absent from the peer-reviewed literature. Search terms and inclusion criteria were further refined in a reflexive manner, incorporating relevant lines of inquiry on wealth indices, risk versus prevalence mapping, and conceptual frameworks for determinants of vaccination. To capture work published since the first search and to follow emergent concepts identified during the initial synthesis, a second systematic search using the same inclusion criteria as the first search was conducted between June and September 2025. This yielded 7 additional records. After screening and full-text assessment of all 63 records, 50 sources were ultimately deemed relevant for inclusion (Figure S1).

### 2.3. Data Charting and Analysis

A data charting form was developed to capture relevant methodological and conceptual elements of each study, structured as a standardised extraction template to support consistent coding across the 55 predefined fields (Table S2). It included the specific spatial approaches, the disciplinary or field orientation, the country context and study design, the main findings related to policy or practice, and the alignment between supporting evidence, methods, and conclusions. Title and abstract screening were conducted by one reviewer. Full-text review and inclusion decisions were led by the same reviewer, with regular consultation and discussion among co-authors to challenge inclusion decisions, clarify conceptual boundaries, and reduce individual selection bias. To assess the consistency of data extraction and interpretation, a second reviewer independently extracted data for a random subsample of 20 included articles. Discrepancies were discussed and resolved through consensus, and the extraction template was refined where needed. This approach is consistent with critical scoping review practice, where transparency and conceptual coherence are prioritised over formal inter-rater reliability metrics.

Our analytical approach combined the descriptive synthesis of methodological trends with a thematic and interpretive synthesis. First, we performed a descriptive summary of the included studies, e.g., how many studies used cluster detection, SAE, MBG or other approaches; which countries or regions were under study; and which data sources were used (household surveys, routine health facility data, etc.). In this descriptive stage, outcome measures were also classified according to whether studies reported overall coverage only or explicitly examined inequities through disaggregation or gap-based metrics (e.g., absolute or relative coverage differences, zero-dose prevalence, or spatial concentration of low coverage).

Next, we conducted a thematic analysis across the studies’ findings and discussions. Through iterative discussions, higher-level thematic categories were created and finalised as: (1) method–to–decision fit (e.g., primary method, intended use case, decision level/spatial scale); (2) data sources and integration (e.g., data sources used, facility-data approach/integration); (3) coverage of determinants: demand-side vs. supply/system factors (e.g., social/behavioural factors and approach; facility-readiness variables); (4) uncertainty and validation practices (e.g., validation approach and metric, prediction-interval width, how uncertainty is presented); (5) decision use, uptake, and co-production (e.g., implementation status, stakeholders involved, actionable recommendations/implementation pathway); and (6) temporal update feasibility (e.g., temporal scale, whether update feasibility is discussed). Outcome measures were classified by metric type: aggregate coverage percentages; coverage gaps (absolute or relative differences between areas or groups); zero-dose or unvaccinated counts/prevalence; spatial clustering indices (e.g., Moran’s I, Getis-Ord Gi, LISA); concentration indices for socioeconomic inequality (e.g., Gini coefficient, Wagstaff index); or exceedance probabilities (probability of falling below a threshold). A study was considered to address immunisation inequities if it used any metric beyond aggregate coverage to identify systematically underserved populations or places, whether defined by socioeconomic characteristics, access constraints, or spatial clustering of low coverage.

These themes were operationalised using the corresponding fields in Table S2. This data extraction process was consistently applied to the 50 sources included in the review, noting that some sources had more extensive published assessments than others.

The results of the critical review are organised as follows. We first describe the spatial methods identified in the literature (MBG, SAE, spatial cluster detection, and other approaches) along with their data sources and methodological limitations. We then examine the degree to which these methods incorporate the multidimensional determinants of immunisation uptake, including alignment with conceptual frameworks such as Andersen’s Behavioural Model and the IA2030 equity agenda. Next, we categorise the intended applications of spatial analyses (campaign micro-planning, routine monitoring, resource allocation, and exploratory research) and assess evidence of actual programmatic uptake. Finally, we discuss technical and institutional challenges for translating spatial outputs into action and identify opportunities to strengthen the link between spatial analysis and immunisation programme decision-making.

## 3. Results

### 3.1. Study Selection and Characteristics of Included Studies

Of the 421 records identified through the initial database search and seven additional records from a follow-up search (Figure S1), 50 studies were included in this review, spanning publication years 2007 to 2025 (with published from 2018 onwards). Table 1 provides an overview of the included studies and their key characteristics, including the study setting, primary spatial analysis approach, and data sources. Since the papers we included were selected intentionally (not randomly or exhaustively), any count or statistics we present, such as the number of studies using a given method, describe only our chosen sample, not the entire body of relevant published research.

**Figure 1:**
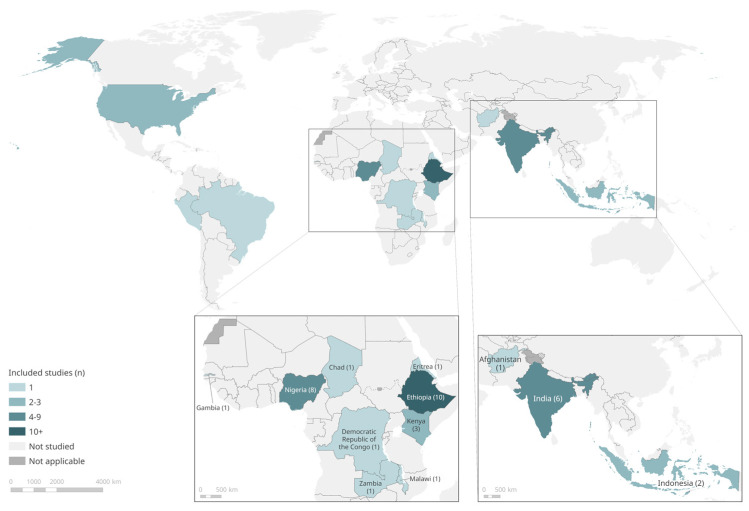
Geographic distribution of the 50 included studies by country. Shading indicates the number of included studies in which each country was analysed, for the 40 studies that focused on a single country. Countries not represented in the included studies are shown in grey. Ten studies adopting a multi-country or regional scope are not shaded individually.

Geographically, the studies covered a broad range of settings (Figure 1). Forty-eight studies (96%) focused on LMICs, with Africa represented in 29 studies (58%), particularly Ethiopia (10 studies) and Nigeria (eight studies). Nine studies examined countries in the South-East Asia or the Eastern Mediterranean Regions, including India (six studies), Indonesia (two studies), and Afghanistan (one study). Two studies were conducted in the Region of the Americas (Peru, Brazil). Two studies analysed high-income settings in the United States (including one focused on Florida). Ten studies took a multi-country or global perspective, analysing data from multiple LMICs simultaneously.

The included studies predominantly relied on cross-sectional ecological designs, defined as analyses using aggregated population-level or area-level data rather than individual-level longitudinal data, to map childhood immunisation coverage and inequalities at subnational levels. This applied to the vast majority of studies (45 of 50), with only a small subset incorporating repeated survey rounds or explicit temporal modelling.

In terms of analytical approaches, coding the primary spatial method for each study revealed a small number of recurring families rather than a wide variety of distinct techniques. We therefore summarise methods in four broad categories: (i) spatial cluster detection and autocorrelation analyses; (ii) SAE and hierarchical models using area-level random effects; (iii) MBG and other continuous-surface mapping approaches; and (iv) other or combined approaches that blended spatial statistics with, for example, machine learning models. Across the 50 studies, 15 studies primarily used MBG or similar Bayesian geostatistical models, 14 studies focused on spatial cluster detection and autocorrelation analyses, 13 studies used SAE approaches (typically hierarchical or multilevel regression models at the area level), and eight studies employed combined or other novel methods (Table 1).

The vast majority of studies relied on household survey data, most commonly the Demographic and Health Survey (DHS), as the primary source of immunisation coverage information (Table 2). Two-thirds of the included articles (34 of 50) made use of DHS data (including country-specific variants such as India’s National Family Health Survey), often drawing on the latest survey or pooling data from multiple rounds. For example, national DHS data were the basis for maps in the Democratic Republic of Congo, Ethiopia, India, Peru, and Nigeria. A small number of studies (4 of 50) sourced data from the UNICEF Multiple Indicator Cluster Survey (MICS), while others drew on country-specific national surveys. Seven studies directly integrated administrative or health system data alongside survey estimates. For example, one analysis in Kenya merged DHS-based coverage data with geocoded health facility locations from the Kenya Master Health Facility List to model geographic accessibility to vaccination services [59]. In Ethiopia, Forzy et al. [52] used District Health Information Software 2 (DHIS-2) administrative coverage data combined with facility assessment data from the Health Resources and Services Availability Monitoring System (HeRAMS). Other studies relied entirely on non-survey sources, including EPI administrative records in Afghanistan [64], surveillance data in Malawi and Eritrea [62,65], and commercial pharmacy data combined with government programme data in the United States [69,72].

### 3.2. Methodological Approaches: MBG, Cluster Detection, SAE, and Others

**Figure 2:**
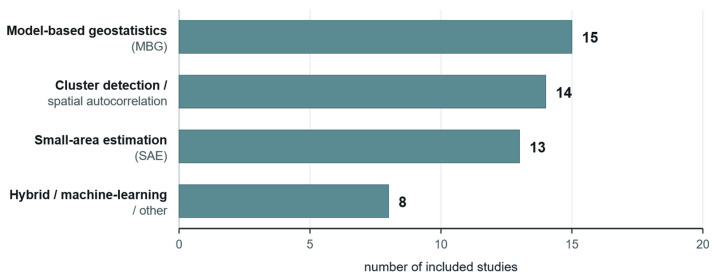
Distribution of the primary spatial analysis method across the 50 included studies.

Fifteen of the 50 included studies (30%) employed an MBG framework (Figure 2), often generating childhood immunisation coverage estimates on regular grids (1 × 1 km or 5 × 5 km), often alongside reporting at administrative levels (e.g., district/state). MBG methods offer several important advantages across spatial grain, data feasibility, uncertainty handling, and interpretability, though with characteristic tradeoffs that recur in the discussion. First, they provide standardised and consistent estimates suitable for regional and cross-border analyses; for example, Mosser et al. [30] produced comparable estimates across 52 African countries, and Sbarra et al. [31] generated estimates for 101 LMICs. Second, because MBG estimates are derived from household survey data rather than administrative records, they avoid the numerator and denominator issues characteristic of routine administrative data. However, surveys carry their own limitations: hard-to-reach populations (including those in conflict-affected, nomadic, or geographically isolated areas) are often underrepresented or excluded from sampling frames, and information bias may arise where vaccination cards are unavailable, and coverage must be estimated from caregiver recall alone. These biases are particularly relevant in fragile settings where MBG estimates might otherwise serve as reference values [41]. Third, the gridded format of MBG outputs offers flexibility: estimates can be aggregated to different geographic units (districts, wards, health facility catchments) and overlaid with environmental, demographic, or socioeconomic data for cross-sectoral health equity analysis [78,80]. In many of these studies, the ability of MBG methods to pinpoint localised pockets of under-vaccination that national or provincial averages can obscure was explicitly emphasised [30,41,59]. Several MBG studies explicitly framed high-resolution mapping as a tool to support campaign planning or microplanning [43,75,78]. Others described their estimates as supporting targeting or prioritisation of underserved areas [41,43,46]. While most MBG studies reported uncertainty intervals and described some form of model validation, typically cross-validation or comparison with held-out survey data, only a small subset explicitly discussed how uncertainty should constrain interpretation or downstream use [30,41,75]. The temporal currency of MBG outputs is constrained by the survey rounds on which they depend.

A limitation of MBG methods is that they require extensive fine-grain covariate data. Unless a set of strongly predictive covariates is included, the model’s interpolations will rely heavily on spatial smoothing and have a high level of uncertainty; if estimates are produced for very fine grids in areas with sparse data, the uncertainty intervals around those estimates may be so large that the point predictions become unreliable for decision-making [32,41,50,75,84]. For instance, Alegana et al. emphasise that “the uncertainties in our estimates highlight areas that require further investigation and higher quality data”. Utazi et al. [32] highlights a different but related point that covariate sets can vary across countries, complicating “one-size-fits-all” global MBG models; they note that “different sets of geospatial datasets were selected for the best model for each of the three test countries[…]challenges may arise in building more universal multi-country or global models”. Another issue is the spatial jittering (or cluster centroid displacement) applied to DHS/MICS cluster coordinates for privacy, which introduces positional error when linking covariates to survey data and can attenuate the strength of covariate associations [85]. These data quality and linkage challenges are not unique to MBG; they affect any spatial method that relies on geolocated survey data and remotely sensed covariates. Furthermore, MBG produces continuous coverage probability surfaces that are not inherently linked to population counts. To translate a coverage probability surface into estimates of how many children are unvaccinated or under-vaccinated in a given pixel or area, one must overlay a corresponding population estimate on the same regular grid as the coverage probability surface [27]. Most MBG studies did this by using gridded population datasets such as WorldPop [30,41], but these population data are themselves modelled estimates with associated uncertainty that no study in our sample propagated to its final estimates of unvaccinated children counts. Finally, MBG assumes that the underlying spatial distribution of vaccination coverage is smooth and continuous; abrupt changes across national borders or local governance discontinuities may be over-smoothed, missing extreme local values.

About one-third of the studies focused on identifying clusters of significantly high or low immunisation coverage in the data. In contrast to MBG, cluster detection methods make minimal data demands and produce outputs that programme staff can read directly, but they offer no flexibility in spatial grain beyond the input units, no model-based uncertainty propagation, and no advantage in temporal currency over the underlying data. Several studies in Ethiopia applied spatial scan statistics to DHS data to flag districts with significantly low coverage [53,71]. The strength of cluster detection studies lies in their simplicity: they highlight where significant clustering of low coverage exists, which can be useful for immediate programme attention. Cluster detection is commonly applied to areal data (e.g., district- or state-level aggregates) rather than to point-referenced observations alone [68,83], and generally cannot incorporate covariates. These methods are most useful when the spatial unit at which clustering is detected coincides with the unit at which programmatic action would be taken.

A subset of studies employed area-level SAE techniques, using hierarchical or multilevel models to “borrow strength” across geographies and improve precision of subnational estimates. The advantage of this approach is interpretability for programme decision-making: outputs are produced directly at the administrative units used for budgeting, accountability, and routine monitoring, and uncertainty is communicated at units where programme staff already act. SAE studies in Nigeria [38] and India [22,58] similarly generated state- or district-level estimates, with Johri et al. [22] applying precision-weighted estimation to produce cluster-level estimates across India’s 707 districts. Mashal et al. [64] applied area-level spatial smoothing to EPI administrative data across Afghanistan’s districts, identifying districts with unexpectedly low uptake. By aligning with established administrative levels, SAE can be readily communicated to policymakers for whom district-, state- or province-level data are highly relevant [86]. The cost of this alignment is that SAE estimates cannot resolve sub-administrative heterogeneity in the way MBG can; where decision-relevant geographies are smaller than the administrative units, SAE will not surface variation MBG could detect.

SAE can improve the precision of direct survey estimates only on the condition that area-level covariates are predictive of the outcome. SAE methodological developments were largely driven by the poverty estimation literature, where auxiliary variables relevant to poverty (income, education, consumption) can be derived from censuses [86]. Equivalent determinants of vaccination (such as maternal education, household wealth, religion, ethnicity, and access to improved water and sanitation) can sometimes be derived from census data, though their availability, temporal alignment, and predictive strength vary considerably across settings. Variables capturing access to related primary health care services, such as antenatal care attendance or institutional delivery rates, were also rarely incorporated as covariates, despite documented associations with vaccination uptake. In the absence of strongly predictive covariates, SAE studies usually rely on the same environmental covariates used in MBG models, averaged at the area-level, potentially diluting finer-scale spatial correlations [87]. Additionally, area-level approaches produce estimates at a level that may mask clustering or inequalities at a finer resolution (for instance, province-level comparisons would mask variations between neighbourhoods).

In a few cases, researchers combined methods or developed alternative approaches (hybrid or machine learning approaches; *n* = 8 of 50 studies). Examples include using learning algorithms (e.g., gradient-boosted regression and generalised additive models) coupled with geospatial modelling to generate 1 × 1 km estimates of vaccination coverage [60]. Dimitrova et al. [49] used a local indicator of spatial association statistics to identify clusters of administrative regions with significantly high or low full immunisation coverage across 43 LMICs, enabling a comparison of subnational heterogeneities and socioeconomic inequalities in childhood vaccination. Wigley et al. [82] similarly flag that their workflow “links together data from a variety of disparate sources, each with their own limitations” including representativeness gaps, where “uncertainties remain as to whether survey samples have been conducted in areas of conflict”. However, none of these studies provided a direct comparison of predictive accuracy or uncertainty between their combined approach and a single-method alternative (e.g., MBG alone), making it difficult to assess whether methodological hybridisation improved performance over standard approaches. Hybrid approaches offer potential gains in predictive accuracy where data are rich but compound the interpretability and uncertainty-attribution challenges already present in MBG. The governance implications of these properties are discussed in Section 3.6.

### 3.3. Data Source Limitations for Spatial Modelling

Nearly all studies relied on household survey data (commonly DHS, MICS, or national health surveys), which remain a good approach for measuring immunisation coverage in LMICs. However, several important limitations stem from this reliance on DHS/MICS data [88]. Firstly, these surveys are typically conducted every 3–5 years and thus cannot capture annual fluctuations or very recent programme efforts. Many analyses assumed that coverage patterns remained relatively stable between survey rounds, or they modelled time trends between survey years in a piecewise fashion. A consequence is that recent programme improvements or declines (for example, a supplementary immunisation activity in a particular province) would not be captured until a new survey is done. A few studies explicitly noted this as a limitation—they could not assess year-to-year changes or the impact of very recent interventions due to data paucity.

Second, household surveys have fixed sample sizes that are optimised for national or, at most, regional representativeness, but direct estimates become increasingly imprecise at finer geographic scales. When estimating coverage for small areas (like districts or ~5 km grids), some areas in the survey have only a handful of sampled children. MBG methods partially address this limitation by drawing strength from all survey clusters when producing estimates for any given location, with more distant clusters having less influence than nearer ones, but estimate uncertainty remains higher in areas where survey clusters are sparse. This leads to wide confidence intervals (i.e., greater uncertainty) for estimates in sparsely sampled areas, though cluster density is not the only determinant of uncertainty; in contexts where overall coverage variability is low, estimates may be relatively precise even with sparse data. In our reviewed studies, it was common to see wider uncertainty bands for areas with fewer clusters sampled (often acknowledged in figures or uncertainty maps). One multi-country analysis, for example, had to aggregate subnational estimates to large administrative divisions in some countries because the survey design did not support finer disaggregation, which complicated cross-country and temporal comparability [49].

Thirdly, data quality issues in surveys can bias results: caregiver recall error and missing vaccination cards may underestimate true coverage or misclassify children’s vaccination status [88,89]. This especially affects indicators like “fully immunised by 12 months” when dates are not recorded. For example, a study in Peru noted that “unfortunately, [the] DHS lacks data on vaccination dates, even when the vaccination card is the source of information,” which hampered their ability to analyse timely vaccination coverage [40]. Surveys also rarely capture doses delivered through campaigns or special outreach, if those are not part of routine card/recall questions. A mapping study in the Democratic Republic of the Congo cautioned that the DHS design made it impossible to know which children had gotten measles vaccine through supplemental campaigns, likely resulting in an undercount of vaccinated children in certain areas [42]; some spatial models might falsely flag areas as “low coverage” simply because the survey did not record campaign doses or because of recall lapses, rather than an actual service delivery gap.

Fourthly, survey sampling frames are typically based on census data that may be several years old and do not account for recent population shifts. This can result in systematic under-representation of mobile or marginalised populations (including urban informal settlements, nomadic communities, and displaced or refugee populations) who are also among those most likely to be missed by immunisation services [75,82].

Another major limitation of using survey data is the absence of health system supply-side variables in most survey datasets. DHS and MICS provide a wealth of information on distal socioeconomic determinants (such as maternal education, household wealth, and urban–rural residence) but rarely capture more proximal, programme-actionable factors such as affordability of vaccination services, caregiver concerns about adverse events, or trust in health workers. They also provide virtually nothing on the accessibility, readiness or performance of health facilities. As a result, most spatial analyses assumed uniform supply or used crude proxies. Many studies in our review did not explicitly incorporate any supply-side factors, effectively treating the immunisation system as uniform and focusing on demand-side factors like maternal education, wealth, or urban–rural residence. Only 9 out of 50 studies (~18%) incorporated substantive supply-side factors, such as facility density, health worker availability, or service readiness data, as opposed to simple travel-time or distance measures derived from facility coordinates. Those that did required linking the household survey and health facility datasets. For instance, Forzy et al. [52] incorporated health-worker staffing levels and health service capacity data from the Health Resources and Services Availability Monitoring System (HeRAMS) to model where service strengthening could most effectively expand access. Additionally, Alegana et al. [41] used modelled travel time to the nearest facility (derived from a friction surface that accounts for terrain, road networks, and land cover) as a covariate when mapping zero-dose prevalence in Chad. Despite these efforts, authors repeatedly noted persistent data gaps: vaccine stockouts, cold chain functionality, and service quality were rarely available at the subnational scale, leaving residual spatial variance unexplained [80]. Factors influencing vaccine acceptance (including hesitancy, which may have sociocultural, informational, or experiential dimensions, as well as religious or community-specific beliefs) were similarly absent from most datasets despite their potential contribution to low coverage in some clusters. Across the board, authors emphasised the need for better data integration (combining surveys with routine health system data) and more frequent or innovative data collection to overcome these limitations in future spatial analyses. Table S4 presents the principal limitations of each major data source alongside concrete remediations, intended as a practical reference for analysts weighing which sources to combine for a given setting and decision type.

### 3.4. Applications and Use-Cases of Spatial Analyses

Four broad applications emerged from our sample of spatial immunisation studies: (1) campaign micro-planning (e.g., targeting supplemental immunisation activities), (2) routine programme monitoring, (3) resource allocation and service design, and (4) exploratory or situational analyses of coverage inequalities (Table 3).

Studies in the first category produced high-resolution coverage maps to guide measles or polio supplementary immunisation activities. For example, Utazi et al. [75] mapped measles vaccination coverage in Nigeria at a fine spatial scale to identify specific low-coverage communities. Comparable hotspot-detection work in Ethiopia [70] and campaign-oriented mapping in Malawi [62] targeted intensified outreach to vulnerable pockets. Of these campaign-oriented studies, only a subset provided explicit documentation of collaboration with implementers; for instance, Kundrick et al. reported measles line-list data compiled in collaboration with the Malawi Ministry of Health and MSF-led campaigns. By contrast, Utazi et al. [75,78] explicitly positioned their outputs for microplanning but did not report confirmed downstream uptake by national programmes. Similarly, Alegana et al. [41] framed the analysis as programmatically relevant, but did not report a specific instance of campaign or microplanning uptake in the paper.

The second category, routine monitoring, generated coverage estimates updated periodically at administrative levels, functioning as a spatially refined scorecard that can also signal where resource allocation needs adjustment. Examples include district-level time series for Zambia [46], high-resolution (1 × 1 km) maps of coverage in Nigeria [60], and continent-wide maps of DTP coverage across Africa [30] or MCV1 across 101 LMICs [31]. These studies are most useful when outputs align with existing health administrative units and reports and when they make uncertainty explicit (e.g., presenting confidence intervals). By doing so, the results can be directly interpreted by decision makers (e.g., a district immunisation officer can see where their district stands relative to targets, and how reliable the estimate is). Indeed, the decision-making level targeted by these studies was often national or subnational, since the goal was to inform internal country monitoring. For example, Sbarra et al. [31] provided country-specific maps intended for national programmes across 101 LMICs, and Kawakatsu et al. [60] focused on subnational planning in Nigeria. At the global level, such estimates also support monitoring of geographic equity indicators under frameworks like IA2030.

The third category integrated household surveys with administrative or facility data layers to inform resource allocation and service design. Studies in Ethiopia, and Kenya incorporated logistics and measures of geographic accessibility (travel time or walking time to immunisation services), and in some cases facility functionality or immunisation service availability, to suggest where to deploy additional resources or service delivery decisions [52,59]. Forzy et al. [52] integrated DHIS-2 administrative immunisation coverage, HeRAMS staffing data, and modelled walking time to predict service strengthening priorities in Ethiopia. Joseph et al. [59] combined survey coverage estimates with modelled travel-time accessibility to identify areas where geographic access barriers contributed to lower immunisation uptake in Kenya. These studies exemplify a more complex data integration approach, and their intended use case was often to inform health system planning decisions at the national or subnational level (e.g., deciding where to add a mobile immunisation team, or which districts should receive additional supply chain support).

The fourth category, exploratory studies, mapped spatial heterogeneity and socioeconomic gradients in coverage without a specified implementation pathway, typically closes with general calls for further research or policy attention rather than specific action plans. Al-Kassab-Córdova et al. [40] mapped subnational heterogeneity in Peru and called for redirected vaccination strategies in low-coverage areas (including vulnerable/rural areas). Geremew et al. [53] identified spatial clustering of measles vaccination in Ethiopia. Similarly, Mashal et al. [64] highlighted security as a key correlate of low immunisation in Afghanistan. Khan et al. [61] paired spatial analysis with broad programmatic recommendations (e.g., strengthening maternal and child services and considering “more innovative models”), rather than detailing a specific implementation plan.

The contrast between the more “operational” studies (those in the first three categories above) and the more “exploratory” studies appears to hinge on a few factors. One key factor is whether the study integrated programmatic data sources in addition to household surveys. Studies that combined surveys with data from immunisation programmes, for example, using post-campaign coverage survey results, routine health information system data, facility surveys (like DHS’ Service Provision Assessments), or known locations of health facilities, tended to produce outputs that were framed as immediately relevant to programme implementation. In some cases, those studies included coauthors from national institutions or partner organisations, which may have facilitated the integration of programmatically relevant data and the framing of outputs for country-level use. For instance, Utazi et al. [78] incorporated recent campaign and health system data in collaboration with national and international partners, resulting in maps and analysis tailored to country planning needs. Joseph et al. [59] in Kenya and Forzy et al. [52] in Ethiopia similarly developed analyses intended for use by local public health teams, yielding findings at the county or district level with potential relevance to local decision-making. However, it is important to note that most studies in our review did not report confirmed programmatic uptake; their operational framing remained theoretical or aspirational.

By contrast, studies that relied solely on large multi-country databases (without direct country stakeholder engagement) were more frequently aimed at global or regional prioritisation and high-level advocacy. For example, Wigley et al. [82] and Dimitrova et al. [49] each analysed dozens of countries together to identify common patterns or outlier regions, informing global discussions on immunisation equity. Yourkavitch et al. [83] and Haeuser et al. [56] produced maps spanning many countries to highlight broad geographic inequalities, intended for use by international initiatives like Gavi or WHO to prioritise support. These studies provided valuable overviews but were not explicitly linked to granular operational planning within any single country. Meanwhile, those single-country studies that were developed by author teams that included individuals from national ministries or in-country teams [75,78] for the most part targeted national or subnational decision-making levels, with results that were more directly applicable to local contexts such as Nigeria’s campaign planning. In summary, we observed that the intended “data-to-decision” pathway of a study was influenced by its data sources and collaborations: analyses that incorporated local health system data and engaged stakeholders were more likely to be framed as yielding actionable insights at the country level, whereas those using harmonised multi-country datasets without direct country engagement were more often exploratory or geared toward global-level policy and strategy. However, the published literature may not fully capture the practical applications of spatial estimates; for instance, coverage estimates from multi-country modelling efforts may be accessed and applied by country programmes or global partners for planning and resource allocation in ways not documented in the original publications.

Both operational and exploratory studies have distinct strengths and limitations. Operational studies maximise immediate relevance and uptake by tailoring outputs to administrative geographies and programme timelines, but they can be constrained by the quality and availability of routine data, may be less generalisable across settings, and sometimes trade methodological novelty for speed and fit-for-purpose reporting. Exploratory studies, in turn, can cover broader geographies, surface emerging patterns, and advance methods under consistent assumptions, yet they may omit critical supply-side or system variables, may not align with the administrative units used for subnational programme decisions, and stop short of implementation guidance.

### 3.5. Alignment with Conceptual Frameworks and Determinants

Within our sample of studies, only a small subset explicitly referenced a formal framework for immunisation equity or immunisation service access when justifying their analysis. For example, one study in Nigeria [39] framed its analysis using Andersen’s behavioural model of health service utilisation, categorising potential determinants of vaccination as predisposing, enabling, or need-based factors. Other studies cited the global IA2030 goals, particularly the emphasis on reaching “zero-dose” children and extending services to the unreached, to justify their equity-oriented mapping [58,70]. Another analysis referenced the Equity Reference Group for Immunisation framework using ERG-identified population groups (e.g., remote-rural, urban poor, and conflict-affected settings) to structure its estimation of zero-dose children across low- and middle-income countries [82]. However, most studies did not explicitly ground their work in any published conceptual framework. In many cases, the analytical approach seemed constrained by data availability rather than guided by a specific theory of change or conceptual model of immunisation equity. Even among the studies that mentioned frameworks, the integration of the framework into the analysis was often limited to background or motivation considerations; operationalising all domains of a framework was typically not possible given the variables available in standard household surveys.

A common theme was that most studies, regardless of stated framework, focused primarily on demand-side socio-demographic factors (which are readily available from household surveys) while supply-side or health system factors had little representation. It is worth noting that the distinction between “demand-side” and “supply-side” factors is not always clear-cut; for example, caregiver perceptions of service availability, accessibility, or quality, which reflect supply-side realities, can be captured through household surveys. The WHO Behavioural and Social Drivers of Vaccination (BeSD) framework’s “practical issues” domain illustrates this approach. However, such measures are not routinely included in DHS or MICS. For example, Johri et al. [58] cite the IA2030 to frame their equity focus, but in practice, their small-area analysis includes standard survey variables (e.g., child and maternal characteristics) with no direct measures of certain IA2030 components, such as service, cold chain functionality, or cold stock management. This gap between conceptual equity frameworks and data availability was common across the studies reviewed: studies analysed the variables available in DHS/MICS (child and maternal characteristics, household wealth, education, place of residence) because these are consistently collected across surveys in standardised formats. Health system factors such as facility density, session availability, vaccine supply continuity, or community engagement were seldom included, not because they are inherently harder to measure, but because they are harder to access: unlike DHS/MICS data which are centrally managed and publicly available, health system data are typically collected and managed locally, may not be standardised across countries, and often have uncertain quality. Incorporating such data requires additional effort in data integration and triangulation, making analyses more resource-intensive and time-consuming.

Overall, there remains a disconnect between the complex, multidimensional equity frameworks, which call for examining factors ranging from behavioural and social determinants to health system performance, and the relatively narrow set of variables that have been mapped in practice. This suggests an opportunity for future work to better incorporate theory-driven variables, for example, incorporating measures of service availability or acceptability, so that spatial analyses of immunisation coverage can comprehensively explain why certain areas and population groups are left behind, not just where they are. Explicitly grounding analyses in immunisation equity frameworks helps strengthen their applications: Andersen’s model [39] clarifies demand-side versus supply-side levers; IA2030’s zero-dose focus [22,82] aligns targeting; and effective coverage logic [90] ensures quality and readiness are accounted for in assessing gaps in immunisation coverage.

### 3.6. Challenges and Opportunities

In our review, we identified both technical and institutional challenges for the use of spatial analysis methods. Rather than cataloguing all challenges exhaustively, we highlight those most pertinent to bridging the gap between spatial outputs and programme decision-making. First, most analyses were only conducted using household surveys, such that (i) supply-side and service-quality determinants are often missing; (ii) sample sizes at fine spatial scales are small, and uncertainty is large; (iii) data collection is infrequent (typically every 3–5 years), and (iv) by the time the gridded estimates are produced, they may already be outdated for routine programme management. Furthermore, most analyses relied on DHS or MICS, rather than the several country-led vaccination coverage surveys that are conducted [91]. These elements partially explain why, despite data quality issues and uncertainties around denominators, most immunisation programmes rely on administrative data for day-to-day operations.

Institutional factors compound these technical limitations. Routine administrative data are embedded in paper-based monitoring systems established over decades and are subject to clear data ownership and governance structures within ministries of health, whereas spatial analyses are comparatively recent and have not yet been systematically integrated into standard programme workflows. Moreover, the caution that programme managers show toward modelled estimates is not simply a matter of unfamiliarity. In official statistics more broadly, a long-standing debate exists between design-based inference, where estimates derive their validity from the sampling design itself, and model-based approaches, whose reliability depends on the assumption that the model is correctly specified [92]. When coverage figures inform resource allocation, accountability frameworks, and public communication, the credibility of the producing institution is at stake [93]. This concern is heightened for immunisation, where public trust in vaccines has proven fragile and where incorrect estimates, whether inflated or deflated, can have immediate consequences for the communities and local officials who must act on them. Transparency in how estimates are produced (including clear communication of model assumptions, validation against local data, and honest characterisation of uncertainty) is therefore not a technical nicety but a precondition for legitimate use in governance. Furthermore, spatial outputs are often produced in formats and at scales that do not align with existing reporting structures, and the resources required to routinely generate, interpret, and act on such outputs have rarely been invested at the district and facility levels where granular information could be most useful. Many of the novel analyses in our review were one-off exercises produced outside programme cycles, with limited local engagement in their design or interpretation. However, because our corpus draws primarily on peer-reviewed and publicly available sources, it likely underrepresents local, operational analyses conducted within ministries of health and partner agencies that are not indexed in bibliographic databases.

These adoption challenges are likely to intensify as the methodological landscape evolves. In our review, 8 of 50 studies employed hybrid or machine learning approaches by combining elements of geostatistical modelling with ensemble methods, gradient boosting, or random forests. While these methods can show gains in predictive performance, they compound the governance concerns outlined above: in conventional spatial models, the assumptions governing estimation (distributional choices, covariance structures, covariate selection) are specified by the analyst and can be scrutinised; in machine learning pipelines, many of these conventions are learned from data, making them less visible to both producers and users of the resulting estimates. When such estimates are intended for use in programme planning or accountability, where local officials must defend decisions based on them, this opacity poses a distinct challenge. There are no established standards for documenting training data provenance and representativeness, for distinguishing sampling uncertainty from algorithmic uncertainty in the published outputs, for benchmarking predictive performance across domains and over successive production cycles, or for maintaining version control when models are retrained. Developing quality frameworks for algorithm-assisted health estimates (ones that move beyond checklists of desirable properties toward operational standards that can be implemented, audited, and compared across settings) should be a priority as these approaches mature.

Despite these challenges, there are clear opportunities to close the technical-institutional gap, which connect directly to global priorities under Gavi 5.0 (2021–2025) and Gavi 6.0 (2026–2030), as well as IA2030, to reach zero-dose children with an enhanced use of geospatial data. Reaching zero-dose children and missed communities is a core priority of Gavi 6.0 strategy, which emphasises more closely integrating zero-dose-related investments with primary health care efforts to advance equity and universal health coverage. One opportunity lies in the use of adaptive spatial sampling to keep maps current between national survey rounds. Instead of treating high-resolution MBG surfaces as endpoints, they can guide where to measure next: intensify sampling where predictive uncertainty is highest, update the surface, and iterate. This active-learning approach, demonstrated in malaria surveillance in Malawi [94] and conceptually applicable to immunisation, offers a practical way to shorten the survey-to-decision cycle, reduce costs, and detect clusters of unvaccinated children developing between national surveys. A second opportunity is a dual-speed data strategy that matches method to decision. Modelled subnational coverage estimates may be more suited to strategic tasks such as multi-year planning, where broad patterns matter more than month-to-month fluctuations. Routine operational management needs more timely data, and household surveys, which typically measure coverage among children aged 12–23 months, inherently reflect vaccination delivered 1–3 years earlier. Here, the emphasis should be on improving and using routine facility reports for day-to-day programme management and monitoring. Rather than replacing routine administrative data, modelled estimates can serve as an independent reference for comparison; countries may also triangulate with other data sources such as targeted subnational surveys (e.g., probability-based or lot quality assurance sampling), vaccine consumption data, or surveillance signals. When modelled coverage estimates and administrative data diverge substantially, the discrepancy can trigger constructive reviews and ground verification. Strengthening denominator estimation with high-resolution population and settlement data is part of the same agenda; integrating geospatial layers can help correct mis-estimated target populations and improve accountability for results [95]. A third lever is co-production: developing analyses in close collaboration with country programme teams. Studies in our review that appeared most oriented toward programmatic application were those pairing analysts with national immunisation programme staff and subnational managers. In this way, the choice of covariates and output geographies mirrored those relevant to immunisation planning (districts, wards, facility catchments) and presented estimates with uncertainty in ways managers could act on [78]. Institutionalising that approach (which entails iterative problem-framing with Expanded Programme on Immunisation staff and subnational managers; template products that include ranked lists with credible intervals and suggested follow-ups; and short trainings on interpreting uncertainty) would allow more maps to have direct relevance to decision-making processes.

### 3.7. Limitations and Strengths of This Scoping Review

Several limitations should be acknowledged. The review was restricted to studies indexed in PubMed and Google Scholar and expert consultation; relevant work in other databases or in languages other than English may have been missed. Consistent with scoping review convention, we did not undertake a formal risk-of-bias appraisal of the included studies, and the analytical judgements presented here reflect our reading of method–decision fit rather than a quantitative appraisal of study quality. Selection was purposive rather than exhaustive, and the counts presented describe our sample rather than the universe of relevant studies. Relatedly, our purposive and iterative selection of studies, appropriate to a critical scoping review, is interpretive rather than fully reproducible, and the substantial narrowing from initial records to included studies reflects judgements informed by the review team’s disciplinary positioning. We regard this as a deliberate trade-off, prioritising depth of methodological appraisal over the exhaustive reproducibility that a systematic review would demand. Finally, evidence of programmatic uptake of spatial outputs was difficult to assess from the published literature alone; while supplementary engagement with WHO, UNICEF, and Gavi colleagues partly addressed this, our characterisation of uptake remains limited to what could be documented in the available materials. Despite these limitations, this review has several strengths. To our knowledge, it is the first critical scoping review to systematically examine the main families of spatial methods as applied to childhood immunisation coverage and equity, and to evaluate them not only on technical merits but on their fit to programme decision-making. Data charting was conducted using a structured 55-field protocol that captured methodological, conceptual, and implementation-relevant dimensions of each study, with a second reviewer independently verifying extraction for a subsample of included studies. The review benefited from sustained engagement with practitioners at the World Health Organisation, UNICEF, and Gavi, the Vaccine Alliance, who contributed grey literature and operational perspectives that are largely absent from the peer-reviewed record.

### 3.8. Key Recommendations

**Figure 3:**
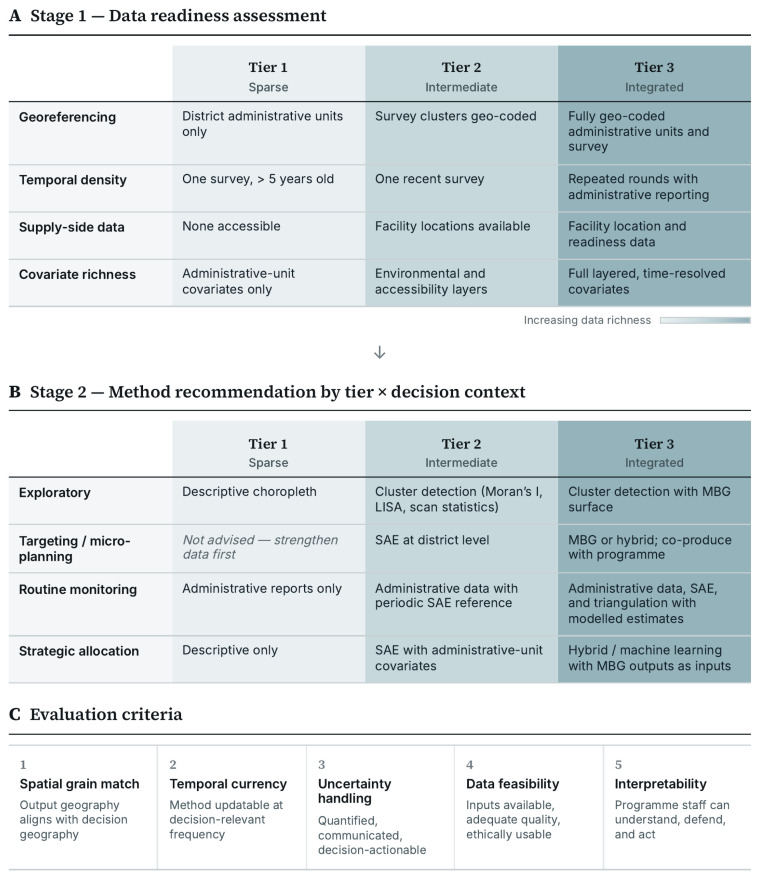
A two-stage framework for selecting spatial methods to inform immunisation programme decisions. Stage 1 (upper panel) characterises data readiness across four dimensions (georeferencing, temporal density, supply-side data, and covariate richness), classifying settings into three tiers from sparse (Tier 1) to integrated (Tier 3). Stage 2 (lower panel) maps recommended method families against the data tier and decision context (exploratory analysis, targeting and micro-planning, routine monitoring, and strategic allocation).

Drawing across the studies reviewed and the analytical themes developed in Section 3.4 and Section 3.5, we summarise five recommendations for the use of spatial methods to advance immunisation equity:

1. Match the method to the decision. MBG may be most useful for sub-district micro-planning; SAE aligns with the administrative units used for budgeting, accountability, and routine monitoring; and cluster detection is the best for rapid, descriptive flagging of anomalously low-coverage areas. Spatial outputs should be selected and presented at the geographic scale at which programmatic decisions are made (Figure 3).

2. Co-produce analyses with national and subnational programme teams. The most operationally relevant studies in our review were those developed in collaboration with Expanded Programme on Immunisation (EPI) staff, including subnational managers, with covariates, output geographies, and uncertainty representations chosen to match programme realities.
3. Adopt a dual-data strategy. Modelled subnational estimates are best suited to multi-year strategic planning, while routine facility data should remain the primary source for day-to-day operational management. The two should be triangulated rather than substituted, with substantial divergences treated as triggers for ground verification rather than as indictments of either source.
4. Use model uncertainty to guide adaptive data collection. Rather than treating high-resolution surfaces as endpoints, programmes can use predictive uncertainty to prioritise where to measure next, shortening the survey-to-decision cycle and detecting clusters of unvaccinated persons developing between national survey rounds.
5. Develop quality frameworks for algorithm-assisted estimates. As machine learning and hybrid approaches become more common, there is an urgent need for operational standards covering training data provenance, the separation of sampling from algorithmic uncertainty, benchmarking across production cycles, and version control. These standards should be auditable and comparable across settings, not merely aspirational checklists.

## 4. Conclusions

In this critical scoping review, we examined the degree to which advanced spatial analytic methods capture inequalities in immunisation, incorporate the multidimensional determinants of immunisation uptake, and inform programmatic decision-making in practice. Across 50 studies, MBG, SAE, cluster detection, and hybrid approaches have successfully exposed subnational inequalities that national averages obscure. The most actionable studies were co-developed with national and subnational teams, integrated programme data, alongside household surveys, and delivered district-ready outputs with explicit next steps. This orientation aligns directly with the equity priorities of IA2030 and Gavi 6.0 (2026–2030).

Important caveats must accompany any expansion of spatial methods in immunisation. First, reliability: modelled estimates remain largely unvalidated, and in data-sparse settings, local-level estimates may appear precise while remaining unreliable for guiding resource allocation. Second, cost: the resources required to develop, validate, and routinely update geospatial models have not been compared systematically against alternatives such as expanded survey sampling, targeted probability-based or lot quality assurance surveys, or strengthened routine administrative data. Third, scale: whether these methods can be sustained within national immunisation programmes, given the technical capacity, institutional arrangements, and recurrent funding required, remains largely untested.

Spatial methods are more likely to contribute to immunisation equity goals not as standalone diagnostic tools, but as components of routine programme workflows: co-produced with local teams, matched to decision-relevant geographies, and updated through ongoing collaborative data collection.

The field’s central unmet challenge is routinisation: whether spatial analysis can be embedded in the recurring workflows of national immunisation programmes and maintained by national teams, rather than one-off academic exercises. Future work should treat the sustainability and routine integration of spatial analysis, and perform a rigorous evaluation of whether it improves decisions over existing data, as the priority. Maps and models are imperfect reflections of the populations they aim to serve, not substitutes for them.

## Figures and Tables

**Table 1:** Summary of included studies (N = 50), with study setting, primary spatial analysis method, immunisation and auxiliary data sources. Supplementary Table S3 includes additional information about each study.

#	Study	Setting	Spatial Method	Immunisation Data	Covariate/Auxiliary Data
1	Aheto (2022) [38]	Nigeria	SAE	Survey (DHS)	WorldPop, MAP
2	Ahmed (2024) [39]	Nigeria	MBG	Survey (DHS)	State-level admin data
3	Al-Kassab-Córdova (2022) [40]	Peru	Cluster detection	Survey (DHS)	—
4	Alegana (2024) [41]	Chad	MBG	Survey (DHS)	WorldPop, AccessMod, GlobCover, SRTM, OSM, HDX facilities
5	Ashbaugh (2018) [42]	DRC	SAE	Survey (DHS)	—
6	Atalell (2022) [43]	Ethiopia	MBG	Survey (DHS)	WorldClim, SRTM, MAP, WorldPop, GLWD
7	Bantie (2024) [44]	Ethiopia	Cluster detection	Survey (DHS)	—
8	Chu (2022) [45]	Indonesia	SAE	Survey (DHS)	—
9	Colson (2015) [46]	Zambia	SAE	Survey (DHS, MICS, LCMS) + Census + Admin	Census, admin data
10	Defar (2019) [47]	Ethiopia	Cluster detection	Survey (Primary)	—
11	Dhalaria (2024) [48]	India	Cluster detection	Survey (NFHS)	—
12	Dimitrova (2023) [49]	43 LMICs	Cluster detection	Survey (DHS)	—
13	Dong (2021) [50]	Nigeria	MBG	Survey (DHS)	Poverty, aridity, nighttime lights, travel time, EVI, urbanicity
14	Endehabtu (2025) [51]	Ethiopia	Cluster detection	Survey (Primary)	—
15	Forzy (2025) [52]	Ethiopia	Other/Combined	Admin (DHIS-2)	DHS, Census, HeRAMS, ESS, WorldPop, electrification
16	Geremew (2019) [53]	Ethiopia	Cluster detection	Survey (DHS)	—
17	Getnet (2025) [54]	48 LMICs	Cluster detection	Survey (DHS)	—
18	Gichuki (2025) [55]	Kenya	MBG	Survey (DHS)	Travel time to facility (MAP)
19	Haeuser (2023) [56]	Multiple countries	MBG	Survey (Country-specific)	WorldPop, GADM, published estimates
20	Holipah (2020) [57]	Indonesia	SAE	Survey (Susenas)	Podes (Village Census)
21	Johri (2021) [58]	India	SAE	Survey (NFHS)	—
22	Johri (2025) [22]	India	SAE	Survey (NFHS)	—
23	Joseph (2020) [59]	Kenya	MBG	Survey (DHS)	Kenya MFL, OSM, SRTM, GlobCover
24	Kawakatsu (2024) [60]	Nigeria	Other/Combined	Survey (DHS, MICS)	WorldPop, TerraClimate, OSM
25	Khan (2018) [61]	India	Other/Combined	Survey (NFHS)	—
26	Kundrick (2018) [62]	Malawi	Other/Combined	Surveillance (Case-based)	WorldPop, Census, MoH facility data
27	Lawal (2023) [63]	Nigeria	SAE	Survey (DHS)	—
28	Mashal (2007) [64]	Afghanistan	SAE	Admin (EPI)	—
29	Mengistu (2023) [65]	Eritrea	Other/Combined	Surveillance (IDSR)	Eritrea Statistics Office, serology lab
30	Mosser (2019) [30]	Africa (52 countries)	MBG	Survey (Country-specific)	GBD estimates, WorldPop, MODIS, environmental
31	Moïsi (2010) [66]	Kenya	MBG	Survey (Primary)	Epi-DSS register, facility survey
32	Pramanik (2015) [67]	India	SAE	Survey (NFHS, DLHS, AHS, CES)	Census
33	Rerolle (2024) [68]	India	Cluster detection	Survey (NFHS)	—
34	Sbarra (2021) [31]	101 LMICs	MBG	Survey (Country-specific)	GBD estimates, WorldPop, environmental
35	Schley (2024) [69]	USA	SAE	Commercial (IQVIA) + Admin (VFC)	SVI, ACS
36	Shiferie (2024) [70]	Ethiopia	Cluster detection	Survey (Primary)	—
37	Tamir (2024) [71]	Ethiopia	Cluster detection	Survey (DHS)	—
38	Tandy (2022) [72]	USA (Florida)	SAE	Admin (Florida Health CHARTS)	ACS, TIGER/Line
39	Tesfa (2022) [73]	Ethiopia	Cluster detection	Survey (DHS)	—
40	Tesfa (2023) [74]	Ethiopia	Cluster detection	Survey (DHS)	—
41	Utazi (2018) [32]	Cambodia, Mozambique, Nigeria	MBG	Survey (DHS)	WorldPop, geospatial covariates
42	Utazi (2020) [75]	Nigeria	MBG	Survey (DHS, MICS, PCCS)	WorldPop, GRID3, geospatial covariates
43	Utazi (2022) [76]	9 LMICs	SAE	Survey (DHS)	ACLED, travel time, WorldPop, urban slum data
44	Utazi (2023) [77]	6 LMICs	MBG	Survey (DHS, MICS)	WorldPop, geospatial covariates
45	Utazi (2024) [78]	Nigeria	MBG	Survey (PCCS)	WorldPop, GRID3, Google Buildings, friction surfaces
46	Utazi (2025) [79]	Nigeria	Other/Combined	Survey (DHS)	WorldPop, travel time, poverty, nightlight, maternal education
47	Wariri (2023) [80]	The Gambia	MBG	Survey (DHS)	WorldPop, geospatial covariates
48	Warren (2017) [81]	Brazil	Other/Combined	Admin (SIH-SUS, NIP)	Live-birth statistics
49	Wigley (2022) [82]	99 LMICs	Other/Combined	Modelled estimates (IHME)	WorldPop, GHS-SMOD, travel time, ACLED, GADM
50	Yourkavitch (2018) [83]	Africa (27 countries)	Cluster detection	Survey (DHS)	DHS Spatial Repository

Notes: MBG = Model-based geostatistics (includes Bayesian spatial models, INLA-SPDE, accessibility modelling), and SAE = small-area estimation (includes multilevel/hierarchical regression).

**Table 2:** Data sources by method.

Method	Survey Only	Admin	Modelled	Total
MBG	15	0	0	**15**
SAE	10	3	0	**13**
Cluster Detection	14	0	0	**14**
Other/Combined	3	4	1	**8**
**Total**	**42**	**7**	**1**	**50**

MBG = Model-based geostatistics (includes Bayesian spatial models fitted via integrated nested Laplace approximation with stochastic partial differential equation spatial fields [INLA-SPDE], accessibility modelling), and SAE = small-area estimation (includes multilevel/hierarchical regression).

**Table 3:** Intended applications of spatial analyses by country.

Country	Campaign Micro-Planning	Routine Monitoring	Resource Allocation /Service Design	Exploratory /Situational	Studies (n)
Afghanistan	–	–	1	–	1
Brazil	–	–	1	1	1
Chad	1	–	1	–	1
DR Congo	–	–	–	1	1
Eritrea	1	–	1	–	1
Ethiopia	4	–	8	2	10
The Gambia	1	–	1	–	1
India	3	–	5	3	6
Indonesia	–	–	2	–	2
Kenya	–	–	2	1	3
Malawi	1	–	1	–	1
Nigeria	3	2	6	2	8
Peru	–	–	1	–	1
United States	–	–	2	–	2
Zambia	–	1	1	1	1
Multi-country/regional	1	3	10	6	10
Total	15	6	43	17	50

## Data Availability

All data supporting the findings of this study are derived from peer-reviewed publications identified through the review’s systematic search strategy. The PRISMA-ScR checklist documenting methodological conformance is provided as Table S1. The protocol governing study selection, data extraction, and synthesis, and the complete data extraction workbook comprising standardised fields across all 50 included studies, are available from the corresponding author upon reasonable request. No new primary data were generated in the course of this research.

## Supplementary Materials

The following supporting information can be downloaded at: https://www.mdpi.com/article/10.3390/vaccines14070572/s1, Figure S1: PRISMA-ScR flow diagram for the identification, screening, and inclusion of studies; Table S1: PRISMA-ScR Checklist; Table S2: Data Extraction Codebook; Table S3: Summary of stated contributions, implementation framing, and programmatic relevance of included studies (*n* = 50); Table S4: Limitations of common data sources for spatial modelling of immunisation coverage and proposed remediations; Section S1: Search strategy.

## Abbreviations

BCG	Bacillus Calmette–Guérin (vaccine)
BeSD	Behavioural and Social Drivers of Vaccination
DHIS-2	District Health Information Software 2
DHS	Demographic and Health Survey
DTP	Diphtheria–Tetanus–Pertussis (vaccine)
EPI	Expanded Programme on Immunisation
ERG	Equity Reference Group for Immunisation
Gavi	Gavi, the Vaccine Alliance
GIS	Geographic Information System
HeRAMS	Health Resources and Services Availability Monitoring System
HIV	Human Immunodeficiency Virus
IA2030	Immunisation Agenda 2030
LISA	Local Indicators of Spatial Association
LMIC	Low- and Middle-Income Countries
LQAS	Lot Quality Assurance Sampling
MBG	Model-Based Geostatistics
MCV1	First Dose of a Measles-Containing Vaccine
MICS	Multiple Indicator Cluster Survey
MSF	Médecins Sans Frontières
PCC	Population–Concept–Context (framework)
PRISMA-ScR	Preferred Reporting Items for Systematic Reviews and Meta-Analyses extension for Scoping Reviews
SAE	Small-Area Estimation
UNICEF	United Nations Children’s Fund
WHO	World Health Organisation
