# Supplementary material for: Assessing Geographic Inequalities in Childhood Immunisation Coverage: A Critical Scoping Review of Spatial Analysis Methods
Source: Vaccines (Basel). 2026 Jun 29;14(7):572. doi: 10.3390/vaccines14070572 (PMC13417178; doi:10.3390/vaccines14070572)
Supplement: Supplementary file 1 [file vaccines-14-00572-s001.zip › vaccines-4295707-supplementary.pdf]

Supplementary Material

Supplementary Figures and Tables

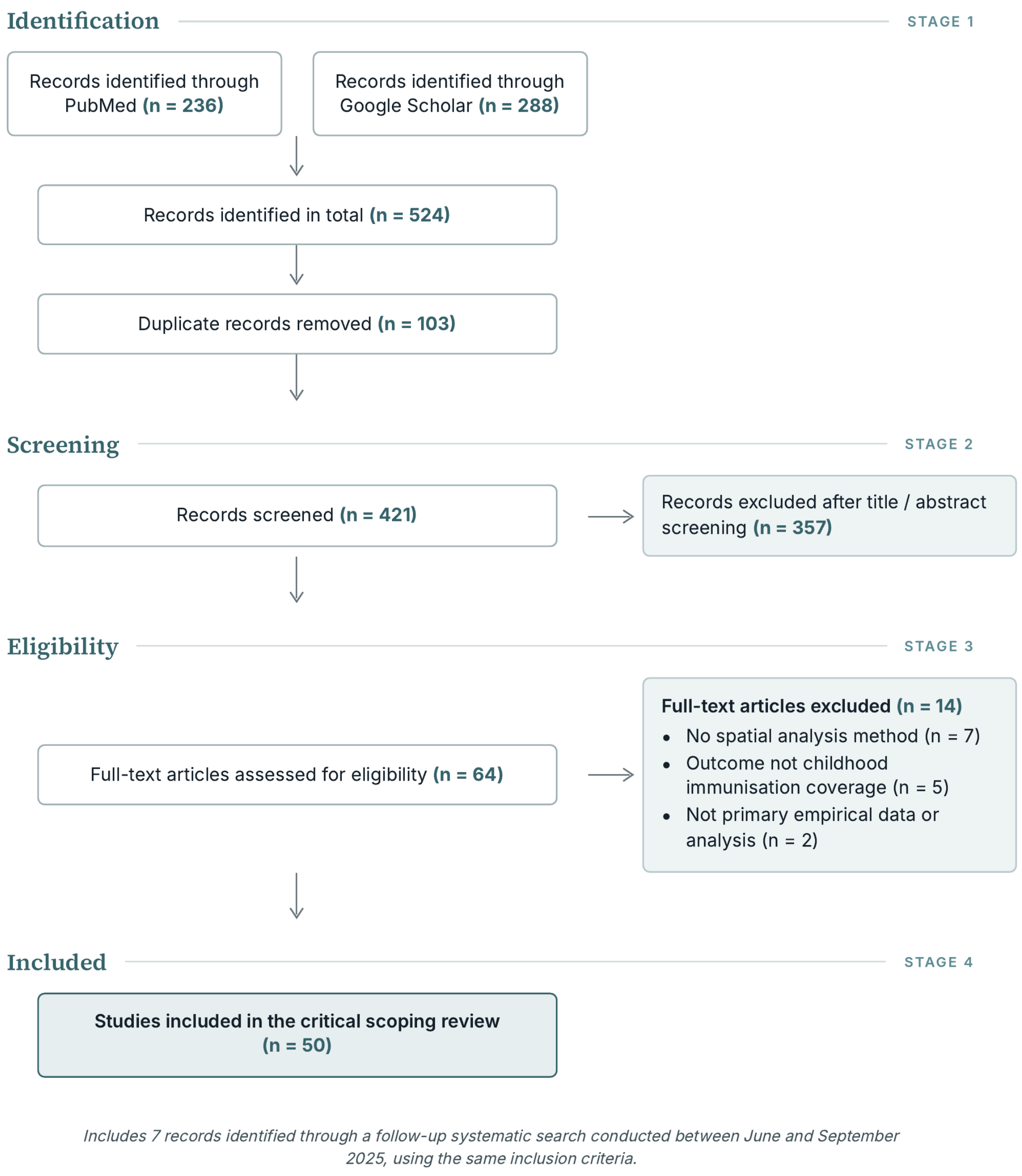

Figure S1: PRISMA-ScR flow diagram for the identification, screening, and inclusion of studies.

Table S1: PRISMA-ScR Checklist

Section	Item	PRISMA-ScR requirement	Status / where reported
Title	1	Identify report as a scoping review	Title & Methods: "critical scoping review"
Abstract	2	Structured summary	Present in abstract
Introduction	3	Rationale	Methods: Study Design and Rationale
Introduction	4	Objectives	Methods: initial questions (i) and (ii)
Methods	5	Protocol and registration	Review not registered (PROSPERO does not accept scoping reviews; no consensus registry for critical scoping reviews). Search strategy, inclusion criteria, and data-charting structure were documented before screening (Supplementary section S1; Table S2).
Methods	6	Eligibility criteria	Methods: inclusion/exclusion criteria
Methods	7	Information sources	Methods: PubMed, Google Scholar; expert/grey inputs
Methods	8	Search	Key terms described in Methods; full strings in S1
Methods	9	Selection of sources	Methods: purposive, iterative; one reviewer screened; interpretive full-text decisions
Methods	10	Data charting process	Methods: template fields; iterative synthesis
Methods	11	Data items	Methods: spatial approach, setting, data sources, policy use, alignment
Methods	12	Critical appraisal of individual sources (optional)	Not conducted (optional for scoping reviews)
Methods	13	Synthesis of results	Methods: descriptive + thematic/interpretive; no meta-analysis
Results	14	Selection of sources of evidence	Results + Figure S1
Results	15	Characteristics of sources	Table 1 (study characteristics)

Section	Item	PRISMA-ScR requirement	Status / where reported
Results	16	Critical appraisal within sources (if done)	Not applicable (optional for scoping reviews)
Results	17	Results of individual sources	Table 1
Results	18	Synthesis of results	Figure 1; Results section
Discussion	19	Summary of evidence	Discussion/Conclusion
Discussion	20	Limitations	Methods & Discussion
Discussion	21	Conclusions/implications	Conclusion section
Funding	22	Funding for the review	Funding section

Table S2: Data Extraction Codebook

This table lists the variables extracted for each included study, grouped by section, with brief descriptions and allowed values or examples. Multiple values for a single field are separated by ' | '. Coding conventions: 'None' = explicitly absent; 'Not mentioned' = not discussed in the paper; 'Not reported' = numeric field without a value provided.

**Field name**	**Section**	**Description**	**Allowed values / examples**
first_author	Study identification	Surname of first author	Text
year	Study identification	Publication year	YYYY
full_citation	Study identification	Complete citation as used in reference list	Text
document_type	Study identification	Type of source	Peer-reviewed | Grey literature | WHO/UNICEF/Gavi doc
disciplinary_orientation	Study identification	Primary disciplinary lens	Epidemiology | Geography | Global Health | Economics | Other
country_region	Study identification	Study location	Country/region names
study_period	Study identification	Time period of data used/analysed	Years or date range
primary_method	Methodology	Primary spatial method applied	Geostatistical Modelling/MBG | Small Area Estimation | Hierarchical/Multilevel Regression | Spatial Cluster Detection |

Field name	Section	Description	Allowed values / examples
			Combined methods | Other
additional_spatial_components	Methodology	Additional spatial methods/components	Spatial smoothing | Kriging | GWR | Spatial autocorrelation (Moran's I) | Hotspot analysis (Getis-Ord Gi*) | SaTScan cluster detection | LISA cluster maps | Cost-surface analysis | Choropleth mapping | Exceedance probability mapping | Spatial interpolation (IDW) | None
primary_aim	Methodology	Main objective as stated by authors	Text
addresses_inequalities	Methodology	Does the study explicitly address inequalities?	Yes | No
inequality_definition	Methodology	Definition/operationalisation of inequality	Text
social_behavioral_factors	Determinants	Social/behavioural factors considered	Caregiver attitudes | Vaccine hesitancy | Cultural factors | Education | Community norms | None
social_behavioral_approach	Determinants	How social/behavioural factors were handled	Directly measured | Proxied | Acknowledged as

Field name	Section	Description	Allowed values / examples
			limitation | Not mentioned
facility_readiness_factors	Determinants	Supply-side factors considered	Vaccine availability | Cold chain | Health workers | Service schedules | Quality indicators | None
facility_data_approach	Determinants	Approach to facility/system data	No facility data | Location/access only | Facility density only | Detailed readiness data | Acknowledged as gap
authors_claim_adequacy	Determinants	Do authors claim determinant coverage is adequate?	Adequate | Partially adequate | Inadequate | Not discussed
adequacy_quote	Determinants	Direct quote regarding adequacy	Quoted text
intended_use_case	Implementation	Intended use of results	Resource allocation | Routine monitoring | Campaign planning | Microplanning | Policy development | Advocacy | Not specified
decision_making_level	Implementation	Level of decision targeted	Global | National | Subnational | Local | Community | Multiple | Not specified
implementation_status	Implementation	Status of use/uptake	Theoretical | Pilot | Routine use | Evidence

Field name	Section	Description	Allowed values / examples
			of uptake | Not discussed
implementation_details	Implementation	Who used it, for what, evidence	Text
routine_vs_campaign	Implementation	Routine or campaign orientation	Routine | Campaign | Both | Not distinguished
implementation_pathway	Implementation	Pathway from evidence to action	Clear | Vague | Evidence of uptake
spatial_scale	Technical details	Spatial resolution of outputs	Grid cell size (e.g., 1x1 km) or administrative level (e.g., District)
temporal_scale	Technical details	Temporal scale/frequency	One-time | Annual | More frequent | Not specified
data_sources	Technical details	Data sources used	Description
data_integration	Technical details	Data integration approach	Survey only | Formal linkage | Spatial overlay | Contextual | Proposed only
integration_barriers	Technical details	Barriers to integration	Technical | Data quality | Institutional | Not discussed
model_diagnostics	Technical details	Model fit/convergence assessment	Detailed | Basic | Not reported | Not applicable
prediction_validation	Technical details	External validation approach	Yes - [method] | None reported

Field name	Section	Description	Allowed values / examples
validation_metric	Technical details	Quantitative validation metric	Numeric value with metric name (e.g., AUROC 0.91, MAE 5.2%)
statistical_software	Technical details	Statistical software/packages used	R-INLA | Stata | SaTScan | ArcGIS | GeoDa | Other
zero_dose_focus	Zero-dose & equity	Explicit zero-dose focus	Yes | Partial | No
equity_dimension	Zero-dose & equity	Primary equity dimension(s)	Geographic | Socioeconomic | Gender | Age | Multiple | Not framed as equity
vulnerable_populations	Zero-dose & equity	Populations identified as at-risk	Text (brief)
ia2030_alignment	Zero-dose & equity	References IA2030/GVAP	Yes | Partial | No
facility_variables_used	Facility variables	Facility-readiness variables used	List
facility_variables_missing	Facility variables	Key missing supply-side variables	List
travel_time_method	Facility variables	Method for estimating travel/access time	Friction-surface | Euclidean distance | Network analysis | None
human_resources	Facility variables	Specialised skills/training noted	Yes/No; brief detail
stock_data	Facility variables	Vaccine stock or cold-chain data used	Yes/No; brief detail

Field name	Section	Description	Allowed values / examples
quote_determinants	Quotes & evidence	Representative quote on determinants	Quoted text (<100 words)
quote_implementation	Quotes & evidence	Representative quote on implementation	Quoted text (<100 words)
quote_limitations	Quotes & evidence	Representative quote on limitations	Quoted text (<100 words)
novel_contribution	Quotes & evidence	Key innovation claimed	Text
methodological_innovation	Quotes & evidence	Novel methodological contribution	Text
evidence_methods_alignment	Alignment checks	Alignment of evidence and methods	Strong | Moderate | Weak
methods_conclusions_alignment	Alignment checks	Alignment of methods and conclusions	Strong | Moderate | Weak
conclusions_policy_alignment	Alignment checks	Alignment of conclusions and policy	Strong | Moderate | Weak
acknowledged_limitations	Alignment checks	Limitations acknowledged by authors	Text
data_quality_discussion	Alignment checks	Discussion of data quality issues	Text
funding_source	Administrative	Main funder/programme impetus	Text
data_governance_notes	Administrative	Data-sharing/ethics/governance notes	Text
reviewer_notes	Administrative	Reviewer comments and flags	Text

Note: During extraction, Hierarchical/Multilevel Regression was coded as a separate primary_method category. For reporting purposes (Table 2 and manuscript text), studies using hierarchical/multilevel regression were grouped under SAE (Small-Area Estimation).

Table S3: Summary of stated contributions, implementation framing, and programmatic relevance of included studies (n = 50)

This table summarises each study's stated main contribution, implementation status, and authors' framing of programmatic relevance, as reported in the original publications. Studies are ordered to match Table 1.

Study	Stated Main Contribution	Implementation Status	Implementation Pathway	Authors' Stated Programmatic Relevance
Aheto (2022)	Examined factors for starting vs completing vaccination series; included geospatial covariates at individual level; used multinomial analysis by evidence source	Theoretical	Vague	"Community-focused interventions, and further research will be required to identify other supply- and demand-side factors as part of an overall strategy to improve childhood vaccination coverage in Nigeria."
Ahmed (2024)	Combined Bayesian multilevel analysis with spatial hotspot mapping to identify state-level clusters of incomplete vaccination in Nigeria; found hotspots concentrated in northern states	Theoretical	Vague	"More efforts are required to improve vaccination sensitization programs and campaigns in Nigeria."

Al-Kassab-Córdova (2022)	Decomposition analysis of 10-year FVC trends in Peru with spatial cluster detection; identified shifting geographic patterns and socioeconomic factors contributing to subnational coverage changes	Theoretical	Vague	"Interventions must be redirected to reduce these geographical disparities."
Alegana (2024)	Fine-scale (1km) zero-dose mapping with age-specific population denominators in fragile setting	Theoretical	Clear	"Improving routine immunization will require investment in the health system as part of enhancing primary health care."
Ashbaugh (2018)	Multilevel analysis of measles vaccination in DRC restricted to card-documented doses to reduce recall bias; identified geographic and socioeconomic predictors of coverage gaps	Theoretical	Vague	"Dated records of measles vaccination must be increased, and groups of children with the greatest need should be targeted."
Atalell (2022)	Spatiotemporal analysis of BCG coverage across 5	Theoretical	Vague	"Maintaining a high level of vaccination

	survey rounds spanning 20 years			coverage across geographical areas is important to prevent TB in Ethiopia."
Bantie (2024)	Identified spatial clusters of low immunisation coverage in Ethiopia using combined hotspot analysis (Moran's I, Getis-Ord Gi*, SaTScan) and multilevel modelling; found significant clustering in Afar and Somali regions	Theoretical	Vague	"It is crucial to reduce disparities in socio-demographic status through enhanced collaboration across multiple sectors and by bolstering the utilization of maternal health care services."
Chu (2022)	Examined age-appropriate vaccination timing for measles and DTwP-3 in Indonesia using multilevel models; found substantial regional variation in timeliness beyond coverage levels	Theoretical	Vague	"These results call for concerted efforts by policy makers to address regional gaps in access to health services and immunization coverage."
Colson (2015)	District-level benchmarking of MCH indicators in Zambia using SAE across 20	Theoretical	Vague	"Subnational benchmarking is important to identify these

	years of survey data; revealed persistent subnational disparities masked by improving national averages			disparities, allowing policymakers to prioritize areas of greatest need."
Defar (2019)	Identified district-level hotspots of low MCH coverage in Ethiopia using spatial autocorrelation and Getis-Ord Gi*; linked geographic clustering to district-level health system factors	Theoretical	Vague	"Identifying district-level factors that influence these outcomes may inform efforts to achieve geographical equitability and universal coverage."
Dhalaria (2024)	Dose-wise spatial analysis of measles vaccination (zero-dose, one-dose, two-dose) mapped by sociodemographic characteristics at district level in India; identified geographic and equity dimensions of incomplete measles vaccination	Theoretical	Vague	"Spatially targeted interventions informed by sociodemographic factors can enhance immunization coverage."
Dimitrova (2023)	Multi-country analysis of subnational heterogeneities and socioeconomic	Theoretical	Vague	"The identified heterogeneities in essential childhood

	inequalities with harmonized metrics			immunization... can be used to inform the design and implementation of localized intervention programs."
Dong (2021)	Proposed improved presentation of MBG coverage maps using discrete colour scales and Average True Classification Probability (ATCP) to better communicate estimate reliability; applied to MCV1 mapping in Nigeria	Theoretical	Vague	"The state-level MCV1 coverage estimates can be used for immunization program monitoring and intervention planning."
Endehabtu (2025)	First application of geographically weighted regression (GWR) to identify spatially varying determinants of zero-dose children in Ethiopia; combined Kriging interpolation with hotspot analysis to map zero-dose prevalence at unmeasured locations	Theoretical	Vague	Identified geographic hotspots of zero-dose children concentrated in pastoral and agrarian regions; determinants (ANC visits, institutional delivery, maternal education) varied spatially,

				suggesting need for regionally tailored interventions
Forzy (2025)	Integration of multiple data sources (DHIS-2, DHS, census, satellite) for district-level immunization prediction in Ethiopia	Theoretical	Clear	"Our modeling could be used... to identify vulnerable woredas based on a limited set of available data."
Geremew (2019)	Combined spatial scan statistics with multilevel modelling in Ethiopia; identified geographic clusters of low MCV1 coverage and associated individual/community determinants including maternal education	Theoretical	Vague	"It is good if the federal ministry of health and other concerned child health programmers give priority of the areas with low MCV1 coverage identified in this study."
Getnet (2025)	Spatial analysis of incomplete basic vaccination hotspots across 48 resource-limited countries; identified significant geographic clustering with hotspots concentrated in sub-	Theoretical	Vague	"The World Health Organization and other stakeholders involved in child healthcare should work together to expand childhood vaccination and prioritize the hotspot areas of

	Saharan Africa and South Asia			developing countries."
Gichuki (2025)	First fine-scale MBG mapping of zero-dose children in Kenya using 2022 KDHS; combined geostatistical modelling with logistic regression examining media exposure and socio-demographic determinants	Theoretical	Vague	Identified hotspots in Tana River, Marsabit, Turkana, and Isiolo; ANC attendance, maternal education, wealth, and travel time to facility were key determinants; recommended enhanced education campaigns and addressing socio-economic barriers
Haeuser (2023)	Framework for assessing spatial overlap between zero-dose and other health indicators to inform integrated service delivery targeting	Theoretical	Vague	"These results provide a framework to assess the potential for joint geographic targeting of interventions."
Holipah (2020)	Longitudinal multilevel analysis of DTP3 coverage trends over 13 years in Indonesia; found that	Theoretical	Vague	"Increasing the number of health centers was not an effective way to increase

	increasing health centre density alone was not associated with improved coverage			immunization coverage."
Johri (2021)	24-year longitudinal analysis of zero-dose children in India with explicit IA2030 framing	Theoretical	Vague	"Interventions that address this cycle of intergenerational inequities should be prioritised."
Johri (2025)	First national small-area (cluster-level) analysis of multiple under-vaccination indicators in India using precision-weighted estimation	Theoretical	Clear	"Achieving India's vaccination goals requires a strategic shift towards identification and targeting of low-immunity clusters at the sub-district level."
Joseph (2020)	National-level analysis in Kenya linking modelled geographic accessibility (travel time to health facilities) to immunisation uptake; identified areas where access barriers contributed most to coverage gaps	Theoretical	Clear	"Strategies that address access barriers in the hardest to reach communities are needed to enhance equitable access to immunisation services in Kenya."
Kawakatsu (2024)	Machine-learning approach (GAMs,	Theoretical	Vague	"High-resolution spatial estimates

	gradient boosting) combining multiple Nigerian surveys to estimate 1x1 km coverage of vaccination and other MCH services; produced grid-level maps for geographic prioritisation			can guide geographic prioritisation and help develop better strategies for implementation plans, allowing limited resources to be targeted to areas with lower coverage."
Khan (2018)	District-level spatial analysis of all routine vaccination components in India using spatial error models; identified persistent geographic disparities across vaccine types	Theoretical	Vague	"Appropriate intervention should be designed to reduce the geographical disparity in the coverage of specific and full immunization across India."
Kundrick (2018)	Comparison of three metrics (VC, susceptible birth cohort, RE) for prioritizing vaccination target areas	Theoretical	Clear	"These methods may be used to allocate effort for prophylactic campaigns or to prioritize response for outbreak response vaccination."

Lawal (2023)	Bayesian zero-inflated Poisson modelling of vaccination dose counts in Nigeria; identified spatiotemporal trends and individual/community-level predictors of under-vaccination across four survey rounds (2003–2018)	Theoretical	Vague	"Improving the uptake of vaccines by educating women on the benefits of hospital delivery and vaccines through radio jingles and posters should be embraced."
Mashal (2007)	Countrywide analysis of immunisation coverage in Afghanistan using multilevel regression with spatial smoothing of EPI administrative data; found significant negative association between insecurity and achievement of 80% coverage targets	Theoretical	Vague	"Security within a country is an important factor for affecting the delivery of immunisation services."
Mengistu (2023)	18-year spatial analysis of measles testing and positivity across all Eritrean sub-zones using surveillance data; identified persistent geographic disparities in measles burden	Theoretical	Vague	"Enhanced surveillance and regional micro planning targeting hard-to-reach areas can be an effective strategy to improve

				measles elimination efforts in Eritrea."
Mosser (2019)	Annual estimates of DTP coverage at 5x5 km resolution across 52 African countries (2000–2016); revealed substantial within-country variation masked by national averages, with persistent low-coverage pockets in Sahelian and conflict-affected zones	Theoretical	Clear	"Local estimates of vaccine coverage can ultimately support more precise targeting of resources to ensure that all children have access to the essential health benefits of vaccination."
Moïsi (2010)	Detailed travel time modeling for vaccine access in rural Kenya using cost-surface analysis	Theoretical	Vague	"The Kenyan EPI reaches nearly all children in Kilifi and delays in vaccination are few, suggesting that vaccines will have maximal impact on child morbidity and mortality."
Pramanik (2015)	Linear mixed model SAE combining five cross-sectional surveys to predict vaccination coverage for 26 Indian	Theoretical	Vague	Found that rural-urban coverage inequality reduced significantly for

	states lacking recent data; model-based estimates closely matched AHS-1 validation; found reduced rural-urban coverage inequality attributed to National Rural Health Mission			most Indian states; attributed gains to National Rural Health Mission supply- and demand-side effects on immunisation in rural India
Rerolle (2024)	Quantified benefits of integrated delivery across vaccines using simulation of Mission Indradhanush-style campaigns	Theoretical	Clear	"Integrated delivery and geographic targeting across core vaccines could accelerate India's progress toward full immunization coverage."
Sbarra (2021)	Comprehensive mapping of MCV1 coverage across 101 LMICs (2000–2019); identified widening urban-rural gaps and subnational inequalities persisting despite national-level improvements	Theoretical	Clear	"Our subnational estimates of routine MCV1 coverage at policy-relevant scales provide a tool for decision-makers to use in advocating for strong, sustainable immunization programmes."

Schley (2024)	Spatial analysis of meningococcal vaccine availability by pharmacy/clinic socioeconomic context and state policy in the US; found school vaccination mandates were key drivers of stocking rates	Theoretical	Vague	"School vaccination strategy was key for improving stocking rates."
Shiferie (2024)	Spatial mapping of zero-dose children in Ethiopia targeting underserved populations; identified hotspot clusters in pastoralist and peripheral regions using primary survey data	Theoretical	Vague	"Implementing routine and mop-up vaccination campaigns in the identified hotspot areas will help Ethiopia to improve coverage and reduce immunization inequalities."
Tamir (2024)	Spatial analysis of IPV coverage in Ethiopia following vaccine introduction in 2015; identified geographic clusters of low uptake and associated determinants including ANC attendance and place of delivery	Theoretical	Vague	"Policies and strategies could benefit from considering antenatal care follow-up, place of delivery, place of residence, and region while implementing inactivated

				poliovirus vaccine immunization."
Tandy (2022)	Spatial regression analysis of non-medical vaccination exemptions at county level in Florida; identified spatial clustering of exemptions and associated socioeconomic predictors	Theoretical	Vague	"Study findings are important in guiding resource allocation for health planning aimed at improving vaccination rates and reducing incidence of vaccine-preventable diseases."
Tesfa (2022)	National-level spatial analysis of MCV1 coverage in Ethiopia; identified hotspot clusters of low coverage concentrated in eastern and peripheral regions	Theoretical	Vague	"Low MCV1 coverage areas should be prioritized to improve vaccination efforts to control measles across the country."
Tesfa (2023)	Spatial and multilevel analysis of complete basic vaccination in Ethiopia using EMDHS 2019; identified geographic clusters and individual/community	Theoretical	Vague	"Developing immunization campaigns targeting areas that had low basic vaccination coverage and designing

	determinants of incomplete vaccination			healthcare programs that can motivate facility-based delivery and ANC follow-up is recommended."
Utazi (2018)	Bayesian geostatistical mapping of age-structured vaccination coverage at 1 km resolution across three LMICs (Cambodia, Mozambique, Nigeria); demonstrated that country-specific covariate sets outperform global models	Theoretical	Vague	"The approaches outlined here provide a route to moving beyond large area summaries of vaccination coverage that mask epidemiologically-important heterogeneities to detailed maps that capture subnational vulnerabilities."
Utazi (2020)	Comparison of routine and campaign MCV coverage at 1 km resolution in Nigeria; identified communities where campaigns failed to close routine immunisation gaps,	Theoretical	Clear	"The results can help to guide the conduct of future campaigns, improve vaccination monitoring and measles

	with explicit zero-dose quantification			elimination efforts."
Utazi (2022)	Multi-country analysis examining ERG priority settings (remote, urban slum, conflict) as predictors of zero-dose/under-vaccination	Theoretical	Vague	"Reaching the Immunisation Agenda 2030 target of reducing the number of zero-dose children by 50% by 2030 will require country tailored analyses and strategies to identify and reach missed communities."
Utazi (2023)	Developed zero-dose vulnerability index integrating multiple mapped risk factors at 1x1 km resolution across six LMICs; compared index-based vs. model-based approaches for classifying district-level vulnerability to under-vaccination	Theoretical	Vague	Demonstrated that integrating mapped risk factors into a composite vulnerability index can improve identification and prioritisation of vulnerable communities; produced district-level classifications for targeting interventions

Utazi (2024)	Integration of building footprint data with health facility catchments to identify areas for fixed vs outreach services; ward and catchment-level zero-dose estimation	Theoretical	Clear	"Our outputs provide a robust evidence base to plan and implement follow-up RI strategies and to guide future campaigns at flexible and operationally relevant spatial scales."
Utazi (2025)	Systematic comparison of geostatistical, ML, and hybrid approaches for vaccination coverage mapping in Nigeria; found geostatistical methods outperformed ML (ANN, BRT) which tended to over-smooth estimates, with implications for spatial prioritisation	Not discussed	Not discussed	"inaccurate identification of priority areas for interventions could result in missing important vulnerable populations, suboptimal resource allocation, reduced impact and persistence of disease circulation or outbreaks"
Wariri (2023)	High-resolution spatial mapping of vaccination timeliness (not just coverage) in The Gambia; bivariate	Theoretical	Vague	"Our approach provides decision-makers with a valuable tool to better understand

	analysis combining late-vaccination prevalence with population density to identify areas of highest absolute burden			local patterns of untimely childhood vaccination and identify districts where strengthening vaccine delivery systems could have the greatest impact."
Warren (2017)	Compared PCV impact on pneumonia hospitalisations across socioeconomic strata in Brazil using synthetic controls and hierarchical Bayesian spatial regression; found significant impact in both low- and high-SES areas	Theoretical	Vague	"These results suggest that PCVs have an important impact on hospitalizations for all-cause pneumonia in both low- and high-income populations."
Wigley (2022)	Global-scale subnational estimation of zero-dose children across 99 LMICs stratified by ERG-defined at-risk settings (remote-rural, urban poor, conflict-affected); quantified zero-dose	Theoretical	Vague	"We demonstrate the need for further inquiry and characterisation of those unvaccinated, the thresholds used to define these, and for more country-

	distribution by vulnerability category			specific and targeted approaches to defining such populations in the strategies and interventions used to reach them."
Yourkavitch (2018)	Cross-border spatial autocorrelation analysis of child health indicators across 27 sub-Saharan African countries; identified transnational clusters of low coverage suggesting shared cross-border drivers	Theoretical	Vague	"Targeting health interventions to high-need populations can be a cost-effective approach to reducing child mortality and can reduce inequities in coverage between the most and least deprived geographic areas."

Table S4. Limitations of common data sources for spatial modelling of immunisation coverage and proposed remediations

Data source	Key limitations	Proposed remediations
**Household surveys (DHS/MICS)**	Conducted every 3-5 years, cannot capture recent change; small samples at fine spatial scale produce wide uncertainty; recall error and missing vaccination cards; sampling frames based on outdated censuses under-represent mobile and marginalised populations; lack supply-side and health-system variables	Integrate with routine/administrative data; adaptive sampling between rounds; strengthen card retention and date recording; update sampling frames with high-resolution population data; add supply-side modules

Data source	Key limitations	Proposed remediations
**Routine / administrative data (HMIS, DHIS-2)**	Numerator and denominator quality issues; inconsistent across districts; locally managed, not standardised; uncertain completeness	Strengthen routine reporting systems; triangulate with modelled estimates; improve denominator estimation with high-resolution population and settlement data
**Health facility / service data (HeRAMS, MFL, SPA)**	Rarely available at subnational scale; cold-chain, stockout, and service-quality data seldom captured; effortful to link to survey data	Invest in facility data integration; geocode facility lists; combine accessibility modelling with coverage estimates
**Modelled population denominators (e.g. WorldPop)**	Themselves modelled with substantial uncertainty; uncertainty rarely propagated to final unvaccinated-child counts	Propagate population uncertainty into final estimates; report combined uncertainty intervals

Supplementary section S1. Search strategy

This review searched one bibliographic database (PubMed/MEDLINE) and one academic search engine (Google Scholar) for studies applying spatial or small-area methods to childhood immunisation coverage. Both searches covered the period from 1 January 2000 to 1 July 2024 and were run on 11 July 2024. No language restriction was applied at the search stage. The full screening and selection flow is reported in the PRISMA flow diagram (Figure S1).

1. PubMed / MEDLINE

Interface: NCBI Entrez E-utilities (programmatic access).

Date searched: 11 July 2024.

Publication date limits: 1 January 2000 to 11 July 2024.

Records retrieved: 236.

Search query:

("spatial analysis"[tiab] OR "spatial statistics"[tiab] OR "geostatistics"[tiab] OR "geospatial"[tiab] OR "small area estimation"[tiab] OR "small-area estimation"[tiab] OR "spatial cluster"[tiab] OR "spatial clustering"[tiab] OR "cluster detection"[tiab] OR "spatial autocorrelation"[tiab] OR "Moran"[tiab] OR "scan statistic"[tiab] OR "Bayesian spatial"[tiab] OR "model-based geostatistics"[tiab] OR "model based geostatistics"[tiab] OR "MBG"[tiab] OR "geographic information system"[tiab] OR "GIS"[tiab]) AND ("immunisation"[tiab] OR "immunization"[tiab] OR "vaccination"[tiab] OR "vaccine"[tiab] OR "vaccines"[tiab] OR "vaccin*"[tiab]) AND ("child"[tiab] OR "children"[tiab] OR "childhood"[tiab] OR "infant"[tiab] OR "infants"[tiab] OR "paediatric"[tiab] OR "pediatric"[tiab] OR "under-five"[tiab] OR "under five"[tiab]) AND ("2000/01/01"[Date - Publication] : "2024/07/31"[Date - Publication])

2. Google Scholar

Access route: SerpAPI Google Scholar engine (programmatic access).

Date searched: 11 July 2024.

Publication date limits: 2000 to 2024 (as_ylo = 2000, as_yhi = 2024).

Records retrieved: 288.

Search query:

("spatial analysis" OR "geostatistics" OR "small area estimation" OR "cluster detection" OR "geospatial") AND (vaccination OR immunization OR immunisation) AND (children OR childhood OR infant)

3. Records retrieved and deduplication

**Source**	**Records**
PubMed / MEDLINE	236
Google Scholar	288
**Total before deduplication**	**524**
Duplicate records removed	103
**Unique records screened**	**421**
